# A likelihood approach to testing hypotheses on the co-evolution of epigenome and genome

**DOI:** 10.1371/journal.pcbi.1006673

**Published:** 2018-12-26

**Authors:** Jia Lu, Xiaoyi Cao, Sheng Zhong

**Affiliations:** Department of Bioengineering, University of California San Diego, La Jolla, California, United States of America; Temple University, UNITED STATES

## Abstract

Central questions to epigenome evolution include whether interspecies changes of histone modifications are independent of evolutionary changes of DNA, and if there is dependence whether they depend on any specific types of DNA sequence changes. Here, we present a likelihood approach for testing hypotheses on the co-evolution of genome and histone modifications. The gist of this approach is to convert evolutionary biology hypotheses into probabilistic forms, by explicitly expressing the joint probability of multispecies DNA sequences and histone modifications, which we refer to as a class of Joint Evolutionary Model for the Genome and the Epigenome (JEMGE). JEMGE can be summarized as a mixture model of four components representing four evolutionary hypotheses, namely dependence and independence of interspecies epigenomic variations to underlying sequence substitutions and to underlying sequence insertions and deletions (indels). We implemented a maximum likelihood method to fit the models to the data. Based on comparison of likelihoods, we inferred whether interspecies epigenomic variations depended on substitution or indels in local genomic sequences based on DNase hypersensitivity and spermatid H3K4me3 ChIP-seq data from human and rhesus macaque. Approximately 5.5% of homologous regions in the genomes exhibited H3K4me3 modification in either species, among which approximately 67% homologous regions exhibited local-sequence-dependent interspecies H3K4me3 variations. Substitutions accounted for less local-sequence-dependent H3K4me3 variations than indels. Among transposon-mediated indels, ERV1 insertions and L1 insertions were most strongly associated with H3K4me3 gains and losses, respectively. By initiating probabilistic formulation on the co-evolution of genomes and epigenomes, JEMGE helps to bring evolutionary biology principles to comparative epigenomic studies.

## Author summary

Epigenetic modifications play a significant role in gene regulations and thus heavily influence phenotypic outcomes. Whereas cross-species epigenomic comparisons have been fruitful in revealing the function of epigenetic modifications, it still remains unclear how the epigenome changes across species. A central question in epigenome evolution studies is whether interspecies epigenomic variations rely on genomic changes *in cis* and, if partially yes, whether different genomic changes have distinct impacts. To tackle this question, we initiated a likelihood-based approach, in which different hypotheses related to the co-evolution of the genome and the epigenome could be converted into probabilistic models. By fitting the models to actual data, each model yielded a likelihood, and the hypothesis corresponded to the largest likelihood was selected as most supported by observed data. In this work, we focused on the influence of two types of underlying sequence changes: substitutions, and insertions and deletions (indels). We quantitatively assessed the dependence of H3K4me3 variations on substitutions and indels between human and rhesus, and separated their relative impacts within each genomic region with H3K4me3. The methodology presented here provides a framework for modeling the epigenome together with the genome and a quantitative approach to test different evolutionary hypotheses.

## Introduction

Milestones of mathematical modeling of DNA evolution were marked by base substitution models in early 1980s [1–3], extension to incorporation of sequence insertions and deletions (indels) in early 1990s [4], and differential treatments of cis-regulatory sequences in the 2000-2010s [5–12]. The rise of interspecies transcriptome comparisons in 2000s [13–16] inspired a series of transcriptome comparison models and evolution models [17–19]. Benefits of joint analysis of interspecies variations of genomes and transcriptomes [20] demanded and eventually led to development of a joint probabilistic evolution model of the genome and the transcriptome [21].

Interspecies epigenome comparisons facilitated discoveries of functions of genomic sequences [22–26]. However, analyses of epigenome evolution remain observational, leading to divergent opinions on the dependence of epigenome conservation on sequence conservation. Some studies reported correlations between genomic and epigenomic changes [27, 28], whereas other studies revealed poor sequence conservation in homologous regions demarcated with the same histone modifications [29–31]. In much shorter timescale, sequence independent passage of histone modifications was observed in multiple generations [32, 33]. The development of evolutionary models for epigenomes would bring mathematical rigor to comparative epigenomics and provide a model competition framework for evaluation of different hypotheses.

In this manuscript, we describe an effort on derivation of the joint probability of a pair of homologous genomic sequences and histone modifications on these sequences. We started with considering four hypotheses, where interspecies epigenomic variations (1) depend only on local sequence substitutions, or (2) depend only on local sequence indels, or (3) depend on both local substitutions and indels, or (4) are independent of local sequence substitutions and indels. We formulated each hypothesis into a probabilistic evolution model and developed a likelihood competition approach for model selection. This model competition approach enabled systematic evaluation of the four evolutionary hypotheses on any homologous sequences.

## Results

Our goal is to develop a probabilistic form of a pair of homologous genomic regions that include the genomic sequences and histone modifications, coupled with each major hypothesis on the co-evolution of genome and epigenome. If we denote the pair of homologous genomic regions as *A* and *B*, our goal is to derive the joint probability *P*(*A*,*B*). For this purpose, we introduce the following notations, model assumptions, and alternative hypotheses on the evolution of genome and histone modifications.

### Notations

We introduce three sets of notations, including indices, observed data, and model parameters. The indices are *h* for indexing histone modifications (*h* = {1,2,…,*H*}), *m* and *n* for indexing nucleotide positions in two DNA sequences, respectively, and *k* for indexing nucleotide positions in a pair of aligned sequences.

The observed data are denoted as follows. *A*^0^,*B*^0^ denote a pair of homologous genomic sequences. *A*^*h*^,*B*^*h*^ denote the states of the *h*^th^ histone modification on *A*^0^,*B*^0^. *A*,*B* denote a pair of homologous regions, including the homologous genomic sequences and the states of each histone modification on these sequences, where *A* = {*A*^0^,*A*^1^,…,*A*^*H*^} and *B* = {*B*^0^,*B*^1^,…,*B*^*H*^}. Let *s*_*A*_ and *s*_*B*_ denote the lengths of *A*^0^ and *B*^0^. Let *a*_0,*m*_ and *b*_0,*n*_ denote the *m*^th^ and the *n*^th^ bases of sequences *A*^0^ and *B*^0^, where *a*_0,*m*_, *b*_0,*n*_ = {*A*,*C*,*G*,*T*}. Let *a*_*h*,*m*_ and *b*_*h*,*n*_ denote the states of the *h*^th^ histone modification at positions *m* and *n* in *A*^*h*^,*B*^*h*^, where *a*_*h*,*m*_ = {0,1} and *b*_*h*,*n*_ = {0,1}. Let $a_{0,k}^{\prime },b_{0,k}^{\prime }$ denote the nucleotides or indels on the *k*^th^ position of an aligned pair of sequences, where $a_{0,k}^{\prime },b_{0,k}^{\prime }=\{A,C,G,T,-\}$. Let $a_{h,k}^{\prime },b_{h,k}^{\prime }$ denote the states of the *h*^th^ histone modification on the *k*^th^ position in a pair of aligned sequences, where $a_{h,k}^{\prime },b_{h,k}^{\prime }=\{0,1,-\}$. Finally, we denote an alignment of two sequences as a *path*, that is $path=\{a_{0,k}^{\prime },b_{0,k}^{\prime }\}$.

The model parameters include *π*_A_,*π*_C_,*π*_T_,*π*_G_, denoting the equilibrium probabilities of the four nucleotide bases. Let $\pi_{1}^{h}$ denote the global equilibrium probability, that is the equilibrium probability of having the *h*^th^ histone modification on any genomic location, and $\pi_{0}^{h}=1-\pi_{1}^{h}.$ Let $\varphi_{A1}^{h}$ denote the local equilibrium probability, that is the probability of having the *h*^th^ histone modification on genomic region *A*, and $\varphi_{A0}^{h}={1-\varphi }_{A1}^{h}$. Denote sequence deletion rate as *μ*, insertion rate as *λ* and substitution rate as *s*. Let *κ*^*h*^ be the rate of switch between 0 and 1, that is installing (0 to 1) or removing (1 to 0) for the *h*^th^ histone modification. Let *t* denote evolutionary time.

### Model assumptions

**Fig 1:**
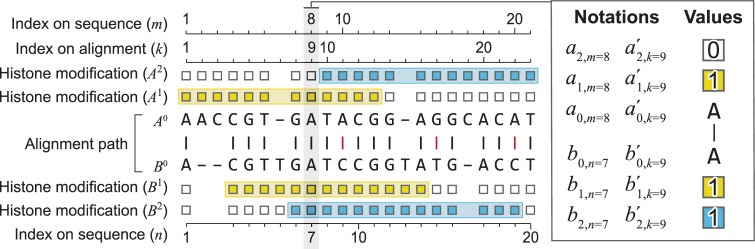
Data types and annotations. A pair of homologous sequences (*A*^0^,*B*^0^) are aligned, where −, | (black) and | (red) are indels, matches and mismatches, respectively. Base locations on each original sequence are indexed by *m* and *n* (indices on sequence). Base locations after sequence alignment are indexed by *k* (indices on path). Peak regions of two histone modifications *A*^1^,*B*^1^, and *A*^2^,*B*^2^ are shown as yellow and blue bands, respectively. A given histone modification on a given sequence, for example *A*^1^, is recorded by binary values on each base, with 1 being inside a peak and 0 being outside the peaks. Insert: notations and values of a specific position. On the 8^th^ position of sequence *A*^0^, the base is A (*a*_0,*m* = 8_ = A). This base becomes the 9^th^ base after alignment ($a_{0,k=9}^{\prime }=A$). This base is inside a peak of the first (yellow) histone modification (*a*_*h* = 1,*m* = 8_ = 1) but outside any blue peaks (*a*_*h* = 2,*m* = 8_ = 0). If we use the base index after sequence alignment (*k*), that include indels, the above notations and values become $a_{1,k=9}^{\prime }=1$ and $a_{2,k=9}^{\prime }=0$.

We assume that the state for each histone modification on each genomic location is binary, that is, *A*^h^ and *B*^h^ are sequences of 0’s and 1’s with the same lengths as *A*^0^ and *B*^0^ (Fig 1). For example, a 5nt sequence of ACGTA (*A*^0^ = ACGTA) that is within an H3K9me3 peak (denote *A*^*h* = *H*3*K*9*me*3^ as *A*^1^) can be written as $\begin{array}{c} A^{0}\\ A^{1} \end{array}=\begin{array}{c} \mathrm{ACGTA}\\ 11111 \end{array}$. For another example, a 10nt sequence ACGTAGGGGG (*B*^0^ = ACGTAGGGGG) with the first 5 bases covered by an H3K9me3 peak and the second 5 bases not covered by any H3K9me3 peak can be written as $\begin{array}{c} B^{0}\\ B^{1} \end{array}=\begin{array}{c} \mathrm{ACGTAGGGGG}\\ 1111100000 \end{array}$, where *B*^1^ denotes the states of H3K9me3. Dependencies of epigenomic states on nearby DNA bases are reflected in the consecutive 1s inside a peak and the consecutive 0s outside peaks. Thus, even though the model we will describe does not explicitly model dependencies of nearby bases, peak calling as the preprocessing step for histone modification data accommodated dependencies of nearby bases. Our second assumption is the widely adopted Pulley principle, namely that genomic evolutionary processes are reversible [3].

### Development of a probabilistic framework for epigenome evolution

**Fig 2:**
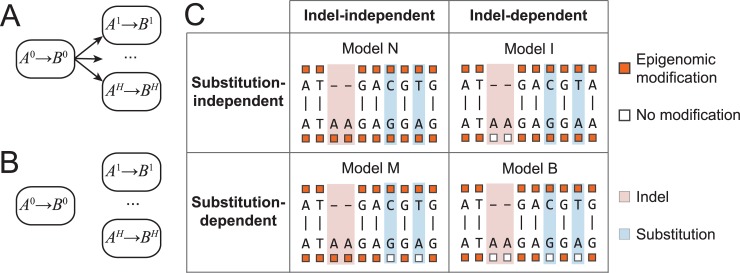
Dependency structures reflecting different evolutionary hypotheses. (A) Interspecies changes of the *h*^th^ histone modification (*A*^*h*^ → *B*^*h*^) depend on local genomic sequence changes (*A*^0^ → *B*^0^). (B) Interspecies epigenomic changes do not depend on local sequence changes. (C) A 2×2 table summarizing assumed dependencies to specific types of sequence changes in each model. Upper (bottom) row: models assuming independence (dependence) of sequence substitutions. Left (right) column: models assuming independence (dependence) of sequence indels.

With the above introduced notations, our goal is to derive *P*(*A*,*B*) = *P*(*A*^0^,*A*^1^,…,*A*^*H*^,*B*^0^,*B*^1^,…,*B*^*H*^), where *A*^0^,*B*^0^ are homologous genomic sequences and *A*^*h*^,*B*^*h*^ (*h* = {1,…,*H*}) are histone modifications on *A*^0^,*B*^0^. To specify such a joint probability, we considered two types of dependency structures. First, descendent genomic sequence depends on ancestral sequence, and histone modifications depend on their underlying genomic sequence. The challenge of using such a dependency structure lies in the lack of complete knowledge of how genomic sequence determines the histone modifications, and therefore generally speaking *P*(*A*^*h*^|*A*^0^) cannot be specified. In the second type of dependency structure, descendent genomic sequence depends on the ancestral sequence, and histone modifications on the descent sequence depend on the histone modifications on the ancestral sequence. Furthermore, the evolutionary changes of each type of histone modification may depend on the underlying genomic sequence changes (Fig 2A) or not (Fig 2B), and conditional on underlying sequence changes the evolutionary changes of different histone modifications are independent of each other (conditional independence) (see Discussion). We elected to specify the joint probabilities with the second type of dependency structure.

Based on the second type of dependency structure, we have

$$P(A,B)=\sum_{path}P(A,B,path)=\sum_{path}P(A,B|path)P(path),$$ (1)

where *path* is an evolutionary path of homologous sequences, corresponding to an alignment of *A*^0^ and *B*^0^ (Fig 1). Any probabilistic expression of sequence alignment can be used for *P*(*path*), and in the work we employ the widely adopted TKF model as the analytical form of *P*(*path*) [4]. *P*(*A*,*B*|*path*) is the probability of observing a pair of homologous sequences and their epigenomes conditional on the sequence alignment. Because all sequence information is contained in *path*, due to conditional independence, we have:

$$\begin{array}{l} P(A,B|path)=P(A^{0},A^{1},\ldots ,A^{H},B^{0},B^{1},\ldots ,B^{H}|path)\\ \;=P(A^{1},\ldots ,A^{H},B^{1},\ldots ,B^{H}|path)\\ \;=\prod_{h=1}^{H}P(A^{h},B^{h}|path). \end{array}$$ (2)

Applying previously introduced notations, we have:

$$\begin{array}{l} P(A^{h},B^{h}|path)=P(B^{h}|path,A^{h})P(A^{h}|path)\\ \;=\prod_{k=1}^{len(path)}P(b_{h,k}^{\prime }|a_{h,k}^{\prime },a_{0,k}^{\prime },b_{0,k}^{\prime })P(a_{h,k}^{\prime }|a_{0,k}^{\prime },b_{0,k}^{\prime }), \end{array}$$ (3)

where len(*path*) is the length of the aligned sequence pair *A*^0^,*B*^0^ (first lane, Fig 1). Taking Eqs (1)–(3) together, we have obtained a probabilistic statement of observing a pair of homologous sequences and their respective histone modifications. Hereafter, we call Eqs (1)–(3) the LCZ model. The LCZ model is fully specified when $P(b_{h,k}^{\prime }|a_{h,k}^{\prime },a_{0,k}^{\prime },b_{0,k}^{\prime })$ and $P(a_{h,k}^{\prime }|a_{0,k}^{\prime },b_{0,k}^{\prime })$ are specified.

### Translation of alternative evolutionary hypotheses into probabilistic models

We restricted this work to considerations of two types of sequence changes, namely substitutions and indels. A total of four possible evolutionary hypotheses can be posed, that are (1) epigenome changes are independent of local sequence changes (Model N), (2) epigenome changes depend on local sequence substitutions but are independent of local sequence indels (Model M), (3) epigenome changes depend on local sequence indels but not local sequence substitutions (Model I), and (4) epigenome changes depend on both local substitutions and indels (Model B, Fig 2C). The four models based on these hypotheses could be summarized as a mixture model of four components. We described the detailed probabilistic form to express each hypothesis and the joint model in Methods and Materials. Furthermore, we will describe a likelihood comparison approach for testing which hypothesis fits actual data, and whether different genomic regions conform to a single evolutionary model.

### Development of an MLE algorithm for parameter estimation

We implemented a maximum likelihood estimation (MLE) algorithm for model fitting. The input data for the MLE algorithm are a list of pairs of homologous regions, hereafter termed *homologous pairs*, each of which contains two homologous sequences, and on each position of each sequence a binary indicator of state of each histone mark. The model parameters include equilibrium probabilities *π* and *φ*, birth and death rates *λ* and *μ*, substitution rate *s*, and the rate of change for each histone modification *κ*^*h*^. Our MLE calculation algorithm was a downhill simplex algorithm. The key for application of downhill simplex algorithm is being able to evaluate the likelihood function with given model parameters, which requires summing over all possible evolutionary paths between the two sequences. This was achieved by dynamic program algorithms (see Methods).

### Evaluation with simulation datasets

We tested performances of the models and the MLE algorithm with simulation data. First, we tested the convergence by comparing the estimated parameters at each iteration with the true parameters (Panel A in S1 Fig). We simulated data with 8 sets of model parameters (S1 Table, Methods) under each of the 4 models (Model M, N, B, I), resulting in a total of 32 datasets. Each dataset contained 100 pairs of 500bp-long homologous sequences and one histone modification on each sequence. We ran the MLE estimation algorithm twice with two initial values on each simulation dataset. Regardless of the initial values, the estimated parameters converged to true values in all simulated datasets (Panel A in S1 Fig), and the negative log-likelihood function decreased monotonically (Panel B in S1 Fig).

For a more comprehensive test, we simulated 10 datasets under each of the 4 models with each of the 8 sets of model parameters (S1 Table), resulting in a total of 320 datasets. For each dataset we ran the MLE algorithm to convergence and quantified the difference between the estimated parameters (*θ*) with true values (θ*) with percent error (*e*), defined as *e* = (*θ*−θ*)/θ* × 100%. We summarized the percent errors from all the simulations for each true value (S2 Fig). Regardless of the true values for *s*, *μ*, *κ*, most of the percent errors of all simulations were contained within 20% ((|*e*| < 20%). Greater variation of *e* was observed when the true values were very small (0.01). As the true values increased to 0.1 or 1, nearly all percent errors were contained within 10% (|*e*| < 10%). We note that the estimated *κ* (rate of H3K4me3 switch) from real data was much larger than 0.1 (S2 Table), and thus in the range where the estimated values nearly always converge to true values.

Next, we tested the capability of identifying the underlying model by comparison of likelihood functions. We generated 5 datasets (columns, S3 Fig) under each hypothesis (Hypothesis M, N, I, or B, S3 Fig), resulting in a total of 20 datasets. For each dataset, we computed the likelihood using every model (Model M, N, I, or B), resulting in four computed likelihoods (four dots in each column, S3 Fig). In all simulation datasets, the model that resulted in the largest likelihood corresponded to the actual hypothesis from which the data were generated, suggesting that the true model corresponding the correct hypothesis could be identified by likelihood comparisons.

### Rates of sequence changes and H3K4me3 change between humans and rhesus monkeys

Our overriding question is whether interspecies changes of histone modifications depend on genomic sequence changes, and whether such dependence is invariant in the entire genome. Toward this goal, we used H3K4me3 changes in primate spermatids as a testbed system. We approached the above question with two major steps. First, we estimated sequence change rates and H3K4me3 change rate, and assessed the sensitivity of these estimates to model assumptions and to data processing procedure. We retrieved public epigenomic data from rhesus macaque and human in round spermatids (GSE68507) [28]. We estimated the sequence change rates (*s*, *μ*) and H3K4me3 change rate (*κ*) from each of the four models. We did not separately provide *λ* in results because *λ* was determined by homologous sequence lengths and *μ* [4]. Our estimation of *s*, *μ*, and *κ* were based on the union of H3K4me3 marked regions [28] and all DNase hypersensitive regions from 95 human cell lines [34], that had a total of 2,824,711 homologous genomic regions. The four models yielded nearly the same estimates for each parameter, where sequence substitution rate *s* was approximately 0.07, deletion rate *μ* was approximately 0.04, and H3K4me3 change rate *κ* was approximately 0.75 (S2 Table). Executing the MLE algorithm 3 times with different initial values converged to nearly the same estimated values. These values were in line with the reports of large amounts of interspecies histone modification changes on homologous sequences, in the same cell type [35]. To assess the sensitivity of these estimates, we re-estimated the parameters with randomly sampled subsets of the homologous genomic regions (S2 Table), and also with re-defined peak regions by applying different thresholds in ChIP-seq peak calling (S3 Table). The estimated parameters by large were insensitive to these alternations, with an expected exception that *κ* exhibited a modest decrease when stringency for peaking calling drastically increased. This was because when few peaks were called from either species (q-value = 0.001, S3 Table), the histone modification would not appear to have changed (no modification in either species).

### Epigenome-to-genome dependency in evolution is not uniform across the genome

**Fig 3:**
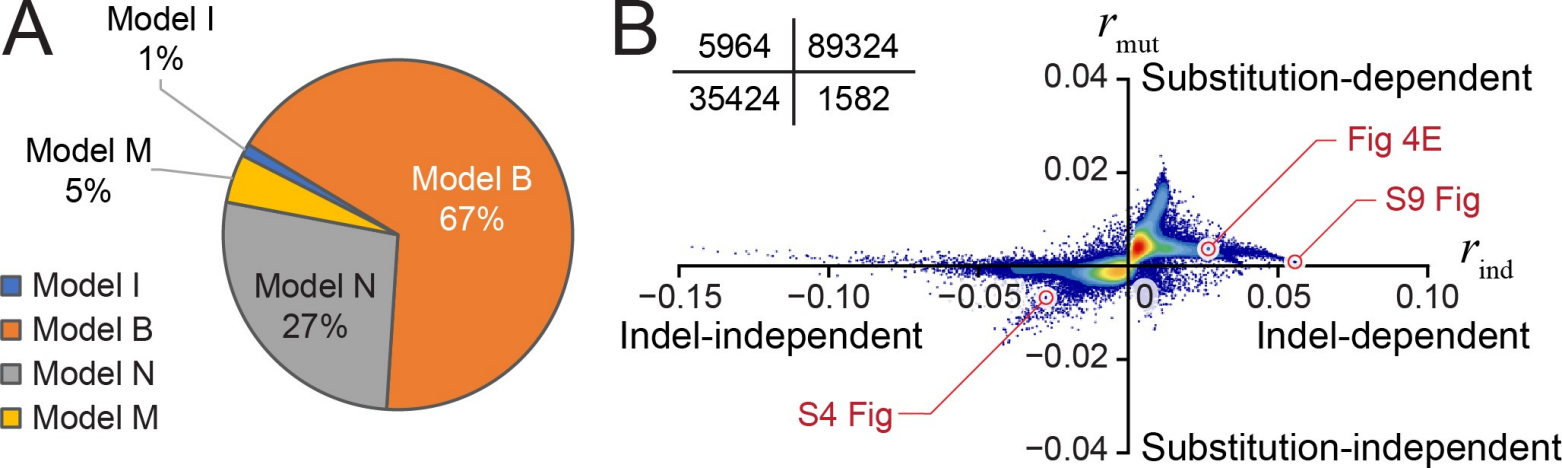
Classifications of homologous genomic regions into four models. (A) Proportions of human-macaque homologous regions classified into each model. (B) Scatterplot of all homologous regions showing the degree of dependence to indels (*r*_*ind*_, x axis) versus the degree of dependence to substitutions (*r*_*mut*_, y axis). Actual data for selected homologous regions (red circles) are given in Fig 4E, S4 and S9 Figs. Insert: the numbers of homologous regions in each quadrant.

Next, we compared the four evolutionary hypotheses on every homologous sequence pair and derived a genome-wide catalogue of the correspondence between genomic region and the best fit evolutionary model. Nearly the entire mappable portion of the human genome (effective genome) has homologous sequence in rhesus macaque genome. Approximately 5.5% of the homologous sequences were covered by H3K4me3 peaks in either species, accounting for 132,294 homologous pairs. For every pair, we computed the likelihood under each of the four models and classified each homologous pair to one of the models according to the largest likelihood. A total of 73% of homologous pairs were classified to Model M, I, or B, where histone modification variation was dependent on local DNA sequence changes (Fig 3A). Most of these homologous pairs were classified to Model B, where histone modification variation was dependent on both local sequence substitution and indel. On the other hand, a total of 27% of homologous pairs were classified with Model N, where histone modification variation did not depend on underlying DNA sequence changes. These results were in line with the idea that the evolutionary changes of the underlying sequences might not completely determine all evolutionary changes of the epigenome.

### Separating contributions of substitutions and indels to epigenome-to-genome dependence

We asked whether sequence substitution or indel better accounts for epigenome-to-genome dependence in evolution. Toward this goal, we derived two metrics *r*_*mut*_ and *r*_*ind*_ to quantify the degrees of dependence of histone changes on substitutions and on indels, respectively (Methods). These metrics were derived from a variation of likelihood-ratio test, where *r*_*mut*_ quantifies the overall fit of a homologous pair to Models N or I (independent of substitutions) versus to Models M or B (substitution dependent), and *r*_*ind*_ quantifies the overall fit to Models N or M (independent of indels) versus to Models I or B (indel dependent). We quantified *r*_*mut*_ and *r*_*ind*_ for every homologous pair and used a scatterplot to visualize the degrees of H3K4me3-to-substitution dependence (*r*_*mut*_, y axis) and H3K4me3-to-indel dependence (*r*_*ind*_, x axis, Fig 3B) of all the analyzed homologous pairs (132,294 in total). Overall, the homologous pairs exhibited greater variations of *r*_*ind*_ than *r*_*mut*_. The majority of homologous pairs exhibited *r*_*mut*_ close to 0, for example 110,400 (83%) homologous pairs exhibited |*r*_*mut*_| < 0.004. Data of these homologous pairs could not clearly infer H3K4me3-to-substitution dependence. A greater number of homologous pairs exhibited non-zero *r*_*ind*_, including 8,965 homologous pairs with *r*_*ind*_ > 0.01, in which H3K4me3 changes were likely attributable to indels. Nearly no homologous pair exhibited H3K4me3 variation that solely depended on substitution (2^nd^ quadrant, Fig 3B), and in some homologous pairs neither substitution or indel appeared to relate to interspecies variation of H3K4me3 (3^rd^ quadrant in Fig 3B, S4 Fig). An alternative normalization method was also used to derive *r*_*ind*_ and *r*_*mut*_, whereas the evaluation of H3K4me3-to-substitution and H3K4me3-to-indel dependencies was not sensitive to different normalization approaches (Methods, S5 Fig).

### Contribution of transposon induced indels to DNA-dependent H3K4me3 changes

**Fig 4:**
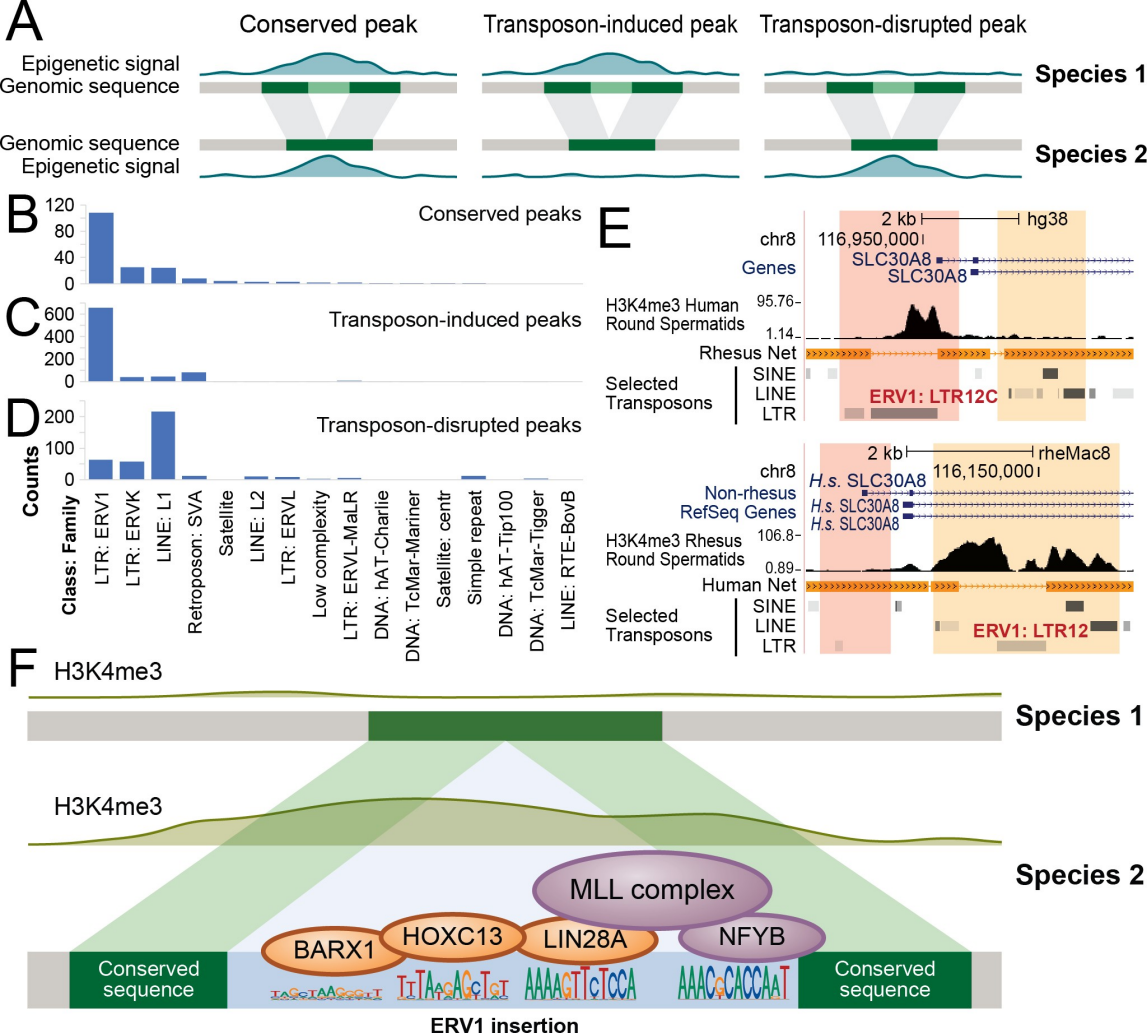
Classes of interspecies covariations of transposons and H3K4me3 peaks. (A) Three classes of covariations of transposon and H3K4me3 peaks. Shaded bands between two species indicate homologous sequences. Light green sequence: insertion of a transposon in Species 1. (B-D) Transposon copy number of each transposon family in conserved peaks (B), transposon-induced peaks (C) and transposon-disrupted peaks (D). (E) A homologous genomic region where interspecies variation of H3K4me3 peaks was associated with ERV1 insertions. Pink bands: a pair of homologous sequences in humans (upper panel) and macaque (lower panel), with a human-specific insertion (ERV1:LTR12C) as well as a human-specific H3K4me3 peak. Orange bands: another pair of homologous sequences, with a macaque-specific copy of ERV1 (ERV1:LTR12) and macaque-specific H3K4me3 peaks. (F) A model for ERV1-induced H3K4me3 peaks. Species specific ERV1 sequence (light blue region in Species 2) harbors motifs for Nfyb, Barx1, Hoxc13 and Lin28a, and induces testis-expressed proteins (orange) to help to recruit Nfyb and the MLL complex (purple).

We asked whether indels induced by different transposon families exhibited similar impacts to interspecies variation of epigenome. To this end, we first classified species-specific transposon insertions into three groups: insertions with no change to H3K4me3 (conserved peak), transposon insertion together with addition (transposon-induced peak) or removal (transposon-disrupted peak) of H3K4me3 (Fig 4A). Next, for each group we identified the number of contributing transposons from every transposon family.

Of the top 10% homologous pairs exhibiting the largest degree of independence between H3K4me3 variations and local indels (4,139 regions, *r*_*ind*_ < −0.0169), 182 (4.4%) contained species-specific transposons. Among these species-specific transposons that did not appear to interfere with H3K4me3, the endogenous retrovirus 1 (ERV1) family of long terminal repeats (LTR) was the most abundant transposon family, accounting for 107 (59%) of the conserved peaks (Fig 4B). This trend did not change when we altered the percentage cutoff of *r*_*ind*_ to 5% and 2% (S6 Fig, S4 Table).

Among the top 10% homologous pairs exhibiting the largest extent of DNA-dependent H3K4me3 changes (9,091 regions, *r*_*ind*_ > 0.099), 854 homologous pairs contained transposon-induced peaks, and 376 homologous pairs contained transposon-disrupted peaks. The ERV1 family was the most abundant transposon family with transposon-induced peaks, accounting for 655 (77%) of all transposon-induced peaks (Fig 4C). The enrichment level of the ERV1 family within transposon-induced peaks was 2.31-fold greater than expectation, with the highest confidence among all transposon types (odds ratio = 2.31, p-value of chi-square test = 1.06 × 10^−6^). This trend did not change when we altered the percentage cutoff of |*r*_*ind*_| to 5% and 2% (S6 and S7 Figs). The promoter region of the human *SLC30A8* gene and its homologous region in macaque was an example in case (Fig 4E). This promoter region harbors two homologous pairs, with one in the upstream regions of the transcription start sites (pink regions, Fig 4E) and the other in the downstream of the TSSs in both species (orange regions, Fig 4E). An ERV1 transposon was inserted in the human upstream region, on which there was a clear H3K4me3 peak, whereas the macaque upstream region did not contain the ERV1 sequence and did not exhibit H3K4me3 (pink regions, Fig 4E). Furthermore, another ERV1 sequence was inserted in the downstream region in macaque where H3K4me3 was installed, whereas the human homologous sequence did not have the ERV1 sequence and did not harbor any H3K4me3 peak (orange regions, Fig 4E).

Unlike transposon-induced peaks that were primarily concentrated to ERV1, transposon-disrupted peaks were primarily contributed from the L1 family, accounting for 213 (57%) of all transposon-disrupted peaks (Fig 4D). The association of the L1 family with transposon-disrupted peaks was 8.60-fold greater than expectation (odds ratio = 8.60, p-value of chi-square test = 5.1 × 10^−22^). These H3K4me3 losses were not likely due to the low mappability of L1 transposable elements, as the flanking regions of the L1 transposons still showed considerably lower H3K4me3 signals compared with their homologous regions after we masked the L1 transposons (S8 Fig). This trend did not change when we altered the percentage cutoff of |*r*_*ind*_| to 5% and 2% (S6 and S7 Figs). A case in point was at the *ZNF630-AS1* promoter, where a L1 transposon (a, S9 Fig) was inserted specifically in the macaque promoter between two ERVL-MaLR family repeats (b, c, S9 Fig), which was coupled with disappearance of H3K4me3. In summary, ERV1, ERVK, L1 and SVA were the most abundant transposons near H3K4me3 peaks. Transposon-induced peaks were most strongly associated with ERV1 transposons, and transposon-disrupted peaks were most strongly associated with L1 transposons.

## Discussion

Current comparative epigenomic studies relied on *ad hoc* analytical methods that were to a considerable extent detached from the core of evolutionary biology. Therefore, a theoretical foundation for comparative epigenomics is in demand. Here, we initiated a class of probabilistic evolutionary models for the genome and epigenome. We provided approaches to explicitly express probabilistic functions of genomic sequences and epigenomic data based on evolutionary hypotheses, thus allowing for data-driven tests of hypotheses of epigenomic evolution. With the methodology, we quantitatively assessed the relative impacts of sequence substitutions and indels to H3K4me3 changes throughout the genome between human and macaque and identified a set of genomic regions where interspecies H3K4me3 changes were primarily attributable to species-specific transposon insertions.

A central question in studies of epigenome evolution is whether interspecies changes of histone modification are independent of evolutionary changes of DNA. If the answer is partially yes, that is, in some specific genomic regions interspecies changes of specific histone modifications are dependent of evolutionary changes of DNA, then the next important question is whether interspecies changes of histone modifications depend on specific types of DNA sequence changes. This second question can be phrased as a set of competing hypotheses, that interspecies changes histone modifications 1) depend only on local sequence substitutions; 2) depend only on local sequence insertions and deletions; 3) depend on both; 4) depend on neither (in which case they may still have a certain degree of dependency on other effects, such as trans effects from sequences, and/or other types of sequence variations).

To test the above hypotheses, the likelihood approach is perhaps the most popular approach, that is to convert each hypothesis into a probabilistic form, and plug in observed data into each probabilistic form, resulting in a likelihood for each hypothesis which is typically interpreted as the compatibility of data to each hypothesis. The hypothesis corresponding to the largest likelihood is considered most supported by observed data. Therefore, we focused this work on how to convert hypotheses into probabilistic forms. Our deliverable, which we called "an evolutionary model" is a mathematical framework to convert each evolutionary hypothesis and multi-species data into a probability. This framework would be applicable to different epigenomic modifications and/or various tissue types/cell lines with corresponding data.

In this work, we used the H3K4me3 variations between human and rhesus as a testbed for the models. We found that a large number of H3K4me3 changes were dependent on local sequence changes. Given that H3K4me3 is usually found overlapping active regulatory elements enriched with transcription binding sites, the dependence might have an association with the alteration of underlying motif sequences. Our models could also be applied to epigenomic marks less associated with regulatory elements to study the dependence between their variations and local sequence changes.

### Gene context for regions with different classification

We asked whether region pairs classified to different models were located in different genomic contexts. For this purpose, we measured the distances between region pairs and their nearest genes. Among the 35,667 regions classified to Model N, 76% and 21% were located within the 5kb flanking regions around transcription start sites (TSSs) of genes in human and rhesus, respectively, suggesting that the majority of regions with H3K4me3 variation not driven by local sequence variations might function as active promoters of their putative target genes. Fewer regions were found close to TSSs in rhesus due to the lack of annotated genes in rhesus monkeys (6,485 genes from the NCBI RefSeq gene annotation). The 96,627 regions with local-sequence-dependent H3K4me3 changes, on the other hand, were considerably farther from TSSs, indicating that epigenomic marks on distal regulatory sequences of genes may be prone to be subject to sequence changes (S10 Fig). The distributions of distance to transcription end sites (TESs) of genes hardly exhibited any differences, as both types of regions were located 5kb to 50kb away from the TESs.

### Epigenome altering transposons possess specific motifs

We identified associations of transposon insertions to both gains and losses of H3K4me3 peaks. While H3K4me3 losses were associated with a variety of transposon families, the ERV1 family of transposons were enriched in H3K4me3 gains. The latter might be a result of species-specific recruitment of transcription factors. In line with this idea, ERV1 was the most notable transposon family involved in species-specific binding of pluripotency regulators OCT4 and NANOG in embryonic stem cells [36]. Our *de novo* motif search revealed a total of 31 DNA motifs that were enriched in ERV1 transposons as compared to other LTRs (Homer p-value < 10^−40^), where the most significant motifs resembled the binding motifs of NFYB (a.k.a. CCAAT box, Homer p-value < 10^−94^), HOXC13 (p-value < 10^−90^), BARX1 (p-value < 10^−89^), and LIN28A (p-value < 10^−84^). According to gene expression data of 37 human tissues from Genotype-Tissue Expression (GTEx) [37] and Human BodyMap 2.0 that were normalized and visualized by Genecards (www.genecards.org), Nfyb was expressed in nearly all human tissues, whereas Hoxc13, Barx1, and Lin28a were all most strongly expressed in testis. Lin28a exhibited 10 times greater expression in testis than in any other analyzed human tissues. The CCAAT box is capable of recruiting ASH2L, a component of the MLL histone methyltransferase complex responsible for H3K4 methylation [38]. These results suggested a model for ERV1 mediated induction of species-specific H3K4me3 in spermatids. ERV1 harbored binding motifs of testis-expressed transcription factors as well as the CCAAT box. Species-specific ERV1 sequences recruited testis-induced HOXC13, BARX1, LIN28A that helped to recruit NFYB and the MLL complex, which in turn established species-specific H3K4me3 peaks (Fig 4F). Finally, the human-specific and macaque-specific insertions of two copies of ERV1 appeared to have induced H3K4me3 in respective insertion regions, near the *SLC30A8* promoter in both species (Fig 4E), providing a potential example of convergent evolution mediated by species-specific transposon insertions.

We also directly compared the motif enrichment between local-sequence-dependent regions and local-sequence-independent regions. The analysis didn’t reveal any motif with enrichment higher than 6% in regions depending on local sequence variations, and there was no clear evidence showing that any motifs were uniquely processed by these regions.

### Epigenome-to-genome dependency between human and mouse

In the comparison between human and rhesus H3K4me3-marked regions, we classified human-rhesus homologous region pairs to different models and separated the effects of substitutions and indels within each region pair. We repeated the analyses between human and mouse, which were more evolutionary distant. We applied the four models to a total of 79,865 human-mouse homologous region pairs with H3K4me3 signals, using public ChIP-Seq datasets from human and mouse round spermatids (GSE68507). 82% region pairs were classified to Model B, I and M, exhibiting local-sequence-dependent H3K4me3 variations. Most of the region pairs (72%) were classified to Model B, where H3K4me3 variations depended on both local sequence substitutions and indels. The rest 18% region pairs were classified to Model N, where H3K4me3 variations were independent of local sequence changes (Panel A in S11 Fig).

Given the farther evolutionary distance between human and mouse, the human-mouse region pairs contained more sequence substitutions compared with the human-rhesus region pairs. Therefore, unlike the human-rhesus region pairs among which the majority showed |*r*_*mut*_| close to 0, the human-mouse region pairs exhibited greater variations of |*r*_*mut*_|, and H3K4me3 variations in most of the homologous region pairs were associated with both substitutions and indels. Nonetheless, similar to that between human and rhesus, we still observed a group of regions in which |*r*_*ind*_| were much larger than |*r*_*mut*_|, meaning that H3K4me3 variations within these regions could be majorly attributed to insertions (Panel B in S11 Fig).

We asked if transposon insertions were also partially responsible for the H3K4me3 variations between human and mouse. Among the top 10% homologous pairs showing the largest extent of insertion-dependent H3K4me3 changes (5,784 regions, *r*_*ind*_ > 0.03), 217 contained transposon-induced peaks, and 678 homologous pairs contained transposon-disrupted peaks. Consistent with the human-rhesus comparison, the L1 family was the most enriched transposon family in transposon-disrupted peaks with an odds ratio of 3.67 (p-value of chi-square test = 1.3×10^−8^) (S12 Fig). Two examples in case were shown in Panels C and D in S11 Fig, in which L1 insertions occurred in mouse and human along with H3K4me3 losses. On the other hand, the ERV1 family exhibited a weaker association with transposon-induced peaks in human and mouse (odds ratio = 1.68, p-value of chi-square test = 0.058) compared with that in human and rhesus. We postulated that this was due to different rates of ERV1 enrichment in human and mouse. Indeed, the ERV1 insertion accounted for 62% transposon-induced peaks in human, whereas only 6.9% in mouse. Another family of the LTR class, ERVK, appeared to be the most abundant transposon family in transposon-induced peaks in mouse, and were found in 55% of the mouse transposon-induced peaks. Interestingly, this was in line with previous transcription factor ChIP-Seq analyses in mouse embryonic stem cells, which revealed that a number of mouse-specific ERVK subfamilies were strongly enriched for multiple TF-binding sites, and might function as cis-regulatory elements [39].

### Locality and causality of epigenome-to-genome dependency

Our models N, I, M and B were designed to evaluate the dependency of the epigenome on the local genomic sequences. If regions showed higher likelihood value in the dependent components of the models, some local effects of sequence evolution upon the epigenetic marker might be inferred, as was shown by the examples in the transposon-related analysis result. It was known that the evolution of sequence itself was affected by multiple factors, such as sequence contexts, transposon insertions and chromatin structures [40–43]. Additionally, sequences changes might also affect the nucleosome positioning, which in-turn influenced the locations of epigenomic marks [44]. Therefore, while some regions showed high dependence on local sequence changes, the underlying biological mechanism for said dependence could be different. However, regardless of the detailed mechanisms of sequence evolution at different locations, their downstream effects, i.e. the sequence change and related epigenomic dependency, would still be captured in our models.

On the other hand, epigenomic variations might also be affected by multiple other sources. Potential mechanisms included trans-acting effects from sequences far from the orthologous regions, such as RNA-DNA interactions [45], possible inheritance of epigenetic states, and other potential factors. The relevant studies, however, were limited to certain molecules and genomic regions. It was unclear if these mechanisms were applicable throughout the genome, and genome-wide data of multiple species were lacking for the purpose of evolutionary studies. Therefore, although the potential effects of these factors were included in the independent-part of our models, it was unlikely to distinguish their contribution due to the lack of prior knowledge and genome-wide data.

Given the modular structure of the framework, the models could be further expanded to incorporate the potential mechanisms in the future when they are better understood. Further distinguishing the underlying mechanism for the epigenome-to-genome dependency and quantitatively describing their relative contributions would be worthwhile efforts in future evolutionary model development.

### Rationale for model assumptions, limitations and potential extensions

We chose to initiate the genome-epigenome joint evolutionary models from base-to-base independence models. This choice was a result from reviewing the history of DNA evolution models. It is now generally accepted that the substitution rate on each DNA base depends on its sequence context, such as inside or outside of codons or transcription factor binding sites. However, DNA evolution models started from modeling each base independently [1–3]. Such seemingly “incorrectness” does not dwarf the paramount importance of the classic models in the history of evolutionary biology. Independence models transformed comparative studies into quantitative analyses, which enabled accumulation of empirical knowledge and eventually fostered development of sequence-context dependent models. Learned from history, although we have also derived sequence-context dependent models on paper, we chose to implement, test, apply, and present the independence model. Our main concern of sequence-context dependent models is the lack of sufficient empirical knowledge at this point of time [46–48]. Implementing dependent models would utilize immature sequence-context assumptions, which without repeated empirical tests and consensus of the field could turn out to be misleading. The initial models presented here, hopefully would equip epigenome comparison with quantitation and thus enable accumulation of empirical knowledge.

Moreover, although these evolution models were independent models, we used widely-accepted procedures during data preprocessing, such as peak-calling for histone-modification-related data sets. Those specific procedures took the dependence among neighboring bases into account. Therefore, when we applied the models, our sample space did not contain any data points contradictory to actual observations (for example, histone modifications that only happened at one or a few intermittent bases or had a pattern not matching any possible nucleosome configuration), as those cases would be excluded by the data preprocessing pipeline. With this constraint, the mathematical approximation of epigenetic modifications in the models, which distributed epigenetic signals to individual nucleotides, would have little effect.

In our models, two types of sequence changes, substitutions and indels, were explicitly considered in the hypotheses. More complex structural variations in the genome, such as inversions, translocations, while not explicitly described in the models, could still be tackled via a multi-step approach: using contemporary comparative genome analysis tools to obtain corresponding syntenies across species first, then applying our models to regions with epigenomic modifications within those syntenies. On the other hand, the dependence between nucleotides could be modeled in future improvements by refining the DNA evolutionary model. Such improvements would provide a more direct approach to modeling epigenome-genome pairs with more complex variations.

As an initial attempt in modeling epigenomic evolution, nucleosome positioning was not included in our models due to the limited availability of nucleosome occupancy datasets, especially the scarcity of comparable nucleosome occupancy data across species [24, 49–51]. In order to incorporate nucleosomes in future expansions, more relevant comparative studies and empirical knowledge are also required so that nucleosome occupancies can be mathematically linked among multiple species, especially in vertebrates.

The current model is limited to two-species comparison. Consequently, genomic and epigenomic gains and losses were not distinguishable between the two species due to the lack of outgroups. Therefore, the extension of the framework to enable multi-species comparison would be a promising goal worth further efforts. This improvement may demand the explicit expression of a joint probability of multispecies (> = 3) DNA sequences and epigenomic modifications, as well as an algorithm for the optimization of the joint probability. The data processing procedure would also need to be expanded for the identification of proper homologous regions shared by multiple species. With outgroups included, sequence mutations may be assigned to specific lineages, and their contribution to the epigenomic variations can be further studied.

Except for the conditional independence assumption, other assumptions in our modeling work were either investigated by prior DNA evolution literature or widely used in analyses of ChIP-seq data, that is, each ChIP-seq peak could be assigned to a beginning and an ending position and DNA inside the beginning and ending positions were considered associated with the histone modification. We foresee two future improvements to further reduce model assumptions. First, in this work we have only considered binary states of histone modifications. To remove this assumption, $a_{h,k}^{\prime }$ and $b_{h,k}^{\prime }$ can be allowed to take any finite discrete numbers, in which case the form of Eq (4) does not change and hence the forms of the rest of the models do not change. Second, the conditional independence assumption can be removed. To model the dependent changes of two histone modifications, for example H3K4me2 and H3K4me3, the two modifications can be coded with the same index (*h*) and let $a_{h,k}^{\prime }\;and\;b_{h,k}^{\prime }$ to take the following form:

$$a_{h,k}^{\prime },b_{h,k}^{\prime }=\{\begin{array}{c} 0,\;H3K4me2=0,H3K4me3=0\\ 1,\;H3K4me2=1,H3K4me3=0\\ 2,\;H3K4me2=0,H3K4me3=1\\ 3,\;H3K4me2=1,H3K4me3=1 \end{array}.$$ (4)

In parallel to the plethora of evidence on DNA-dependent installation and removal of histone modifications, a smaller but increasing amount of data suggest trans-generation DNA-independent inheritance of histone modifications [32, 33]. It remains unclear how many generations could DNA-independent epigenetic inheritance endure, or more importantly whether it is preserved in evolutionary timescale. By initiating probabilistic models of epigenome-genome evolution, this work begins to offer a quantitative framework to test different hypotheses related to epigenomic evolutionary changes. With the expansion of knowledge, more relevant hypotheses can be incorporated into the framework, which may enable the comparison of the contribution of cis-effects, trans-effects and transgenerational inheritance, and hence help to address the questions above. Future developments of epigenome-genome evolution models may also begin to address questions including whether any evolutionary selection acts on the epigenome independently of the genome, and whether any selection forces were received jointly by genome and epigenome. Therefore, we anticipate integrated analyses of genome-epigenome data to expand the domain of evolutionary biology, and the development and deployment of epigenome-genome evolution models to be essential for this expansion.

## Materials and methods

### Modeling dependencies of epigenomic changes on sequence mutations

#### Model N

Model N assumes that epigenomic changes are independent of both substitutions and indels (Model N, Fig 2). We model evolutionary process of epigenomic changes as a Poisson process, in which the transition probability in time *t* is:

$$g_{a_{h,k}^{\prime }{,b}_{h,k}^{\prime }}(t)=\{\begin{array}{c} e^{-\kappa^{h}t}+\pi_{b_{h,k}^{\prime }}^{h}(1-e^{-\kappa^{h}t}),\;a_{h,k}^{\prime }=b_{h,k}^{\prime }\\ \pi_{b_{h,k}^{\prime }}^{h}(1-e^{-\kappa^{h}t}),\;a_{h,k}^{\prime }\neq b_{h,k}^{\prime } \end{array},$$ (5)

where $a_{h,k}^{\prime }$ and $b_{h,k}^{\prime }$ are binary states of the *h*^th^ histone modification on the *k*^th^ position of an alignment, and $\pi_{b_{h,k}^{\prime }}^{h}$ is the equilibrium probability of having the *h*^th^ histone modification on the *k*^th^ position, namely $\pi_{1}^{h}=P(b_{h,k}^{\prime }=1)$ and $\pi_{0}^{h}=P(b_{h,k}^{\prime }=0)$. This probabilistic form is similar to the substitution model of DNA evolution [3]. On a position without indel ($a_{0,k}^{\prime }\neq "-"\;\mathrm{and}\;b_{0,k}^{\prime }\neq "-"$), we have:

$$P(b_{h,k}^{\prime }|a_{h,k}^{\prime },a_{0,k}^{\prime },b_{0,k}^{\prime })=g_{a_{h,k}^{\prime }{,b}_{h,k}^{\prime }}(t),$$ (6)

and

$$P(a_{h,k}^{\prime }|a_{0,k}^{\prime },b_{0,k}^{\prime })=\pi_{a_{h,k}^{\prime }}^{h}\times g_{a_{h,k}^{\prime }{,a}_{h,k}^{\prime }}(0)=\pi_{a_{h,k}^{\prime }}^{h}.$$ (7)

In order to model epigenomic changes on insertions and deletions, we introduce four parameters $\varphi_{A0}^{h},\;\varphi_{A1}^{h}$ and $\varphi_{B0}^{h},\;\varphi_{B1}^{h}$ to represent local equilibrium probabilities of epigenomic states 0 and 1 in genomic regions *A* and *B*, respectively ($\varphi_{A0}^{h}={1-\varphi }_{A1}^{h}$ and $\varphi_{B0}^{h}={1-\varphi }_{B0}^{h}$). Unlike global equilibrium probabilities $\pi_{0}^{h}$ and $\pi_{1}^{h}$, which are estimated from all homologous regions in the entire genomes, local equilibrium probabilities are estimated from each genomic region. On an insertion in the descendent sequence ($a_{0,k}^{\prime }="-"\;and\;b_{0,k}^{\prime }\neq "-"$), the transition probability is modeled as a mixture of the two transitions:

$$P({b_{h,k}^{\prime }|a}_{h,k}^{\prime },a_{0,k}^{\prime },b_{0,k}^{\prime })=\varphi_{A0}^{h}g_{0,b_{h,k}^{\prime }}(t)+\varphi_{A1}^{h}g_{1,b_{h,k}^{\prime }}(t),$$ (8)

where $g_{0,b_{h,k}^{\prime }}(t)$ is the transition probability from the state 0 to the observed state $b_{h,k}^{\prime }$, and $g_{1,b_{h,k}^{\prime }}(t)$ is the transition probability from the state 1 to the observed state $b_{h,k}^{\prime }$. Because there is no place for histone mark on the *k*^th^ position in the ancestral sequence ($a_{h,k}^{\prime }$ is not observed), we denote:

$$P(a_{h,k}^{\prime }|a_{0,k}^{\prime },b_{0,k}^{\prime })=1.$$ (9)

On a deletion in the descendent sequence ($a_{0,k}^{\prime }\neq "-"\;\mathrm{and}\;b_{0,k}^{\prime }="-"$), based on the reversibility of the evolutionary process we model the transition as a mixture of two transitions:

$$P(a_{h,k}^{\prime }|a_{0,k}^{\prime },b_{0,k}^{\prime })=\varphi_{B0}^{h}g_{0,a_{h,k}^{\prime }}(t)+\varphi_{B1}^{h}g_{1,a_{h,k}^{\prime }}(t).$$ (10)

For the completeness of the model, we denote:

$$P(b_{h,k}^{\prime }|a_{h,k}^{\prime },a_{0,k}^{\prime },b_{0,k}^{\prime })=1.$$ (11)

Taken together, $P({b_{h,k}^{\prime }|a}_{h,k}^{\prime },a_{0,k}^{\prime },b_{0,k}^{\prime })$ and $P(a_{h,k}^{\prime }|a_{0,k}^{\prime },b_{0,k}^{\prime })$ are given by:

$$P({b_{h,k}^{\prime }|a}_{h,k}^{\prime },a_{0,k}^{\prime },b_{0,k}^{\prime })=\{\begin{array}{cc} g_{a_{h,k}^{\prime }{,b}_{h,k}^{\prime }}(t), & a_{0,k}^{\prime },b_{0,k}^{\prime }\neq "-"\\ \varphi_{A0}^{h}g_{0,b_{h,k}^{\prime }}(t)+\varphi_{A1}^{h}g_{1,b_{h,k}^{\prime }}(t), & a_{0,k}^{\prime }="-"\;and\;b_{0,k}^{\prime }\neq "-"\\ 1, & a_{0,k}^{\prime }\neq "-"\;and\;b_{0,k}^{\prime }="-" \end{array},$$ (12)

$$P(a_{h,k}^{\prime }|a_{0,k}^{\prime },b_{0,k}^{\prime })=\{\begin{array}{cc} \pi_{a_{h,k}^{\prime }}^{h}, & a_{0,k}^{\prime },b_{0,k}^{\prime }\neq "-"\\ \varphi_{B0}^{h}g_{0,a_{h,k}^{\prime }}(t)+\varphi_{B1}^{h}g_{1,a_{h,k}^{\prime }}(t), & a_{0,k}^{\prime }\neq "-"\;and\;b_{0,k}^{\prime }="-"\\ 1, & a_{0,k}^{\prime }="-"\;and\;b_{0,k}^{\prime }\neq "-" \end{array}.$$ (13)

At this point, all the terms in the LCZ model have been specified. Eqs (12) and (13) specify Model N, where epigenomic changes are independent of sequence changes.

#### Model M

In this model, epigenomic changes are dependent of sequence substitutions but independent of indels (Model M, Fig 2). On a matched (no substitution) base at the *k*^th^ position in the alignment, namely $a_{0,k}^{\prime }=b_{0,k}^{\prime }$, the epigenomic change is modeled with the Poisson process $g_{a_{h,k}^{\prime }{,b}_{h,k}^{\prime }}(t)$ (Eq (4)), whereas on a position with substitution ($a_{0,k}^{\prime }\neq b_{0,k}^{\prime }$ and $a_{0,k}^{\prime },b_{0,k}^{\prime }\neq "-"$), the descendent epigenomic state is modeled by global equilibrium probabilities,

$$P(b_{h,k}^{\prime }|a_{h,k}^{\prime },a_{0,k}^{\prime },b_{0,k}^{\prime })=\pi_{b_{h,k}^{\prime }}^{h}.$$ (14)

On indels, the epigenomic changes is modeled with the same approach as in Model N. Taken together, Model M is specified as:

$$P({b_{h,k}^{\prime }|a}_{h,k}^{\prime },a_{0,k}^{\prime },b_{0,k}^{\prime })=\{\begin{array}{cc} g_{a_{h,k}^{\prime }{,b}_{h,k}^{\prime }}(t), & a_{0,k}^{\prime }=b_{0,k}^{\prime }\\ \pi_{b_{h,k}^{\prime }}^{h}, & a_{0,k}^{\prime }\neq b_{0,k}^{\prime }\;and\;a_{0,k}^{\prime },b_{0,k}^{\prime }\neq "-"\\ \varphi_{A0}^{h}g_{0,b_{h,k}^{\prime }}(t)+\varphi_{A1}^{h}g_{1,b_{h,k}^{\prime }}(t), & a_{0,k}^{\prime }="-"\;and\;b_{0,k}^{\prime }\neq "-"\\ 1, & a_{0,k}^{\prime }\neq "-"\;and\;b_{0,k}^{\prime }="-" \end{array},$$ (15)

$$P(a_{h,k}^{\prime }|a_{0,k}^{\prime },b_{0,k}^{\prime })=\{\begin{array}{cc} \pi_{a_{h,k}^{\prime }}^{h}, & a_{0,k}^{\prime },b_{0,k}^{\prime }\neq "-"\\ \varphi_{B0}^{h}g_{0,a_{h,k}^{\prime }}(t)+\varphi_{B1}^{h}g_{1,a_{h,k}^{\prime }}(t), & a_{0,k}^{\prime }\neq "-"\;and\;b_{0,k}^{\prime }="-"\\ 1, & a_{0,k}^{\prime }="-"\;and\;b_{0,k}^{\prime }\neq "-" \end{array}.$$ (16)

#### Model I

In this model, epigenomic changes depend only on sequence indels but not on substitutions (Model I, Fig 2). On a position that is not indel ($a_{0,k}^{\prime }\neq ”-“\;\mathrm{and}\;b_{0,k}^{\prime }\neq ”-“$), we use the same Poisson process (Eq (5)) as that in Model N to model the epigenomic changes.

On an insertion in the descendent sequence ($a_{0,k}^{\prime }="-"\;and\;b_{0,k}^{\prime }\neq "-"$), the transition probability becomes invariant of *t*:

$$P(b_{h,k}^{\prime }|a_{h,k}^{\prime },a_{0,k}^{\prime },b_{0,k}^{\prime })=\pi_{b_{h,k}^{\prime }}^{h},$$ (17)

and since $a_{h,k}^{\prime }$ is not observed, we denote:

$$P(a_{h,k}^{\prime }|a_{0,k}^{\prime },b_{0,k}^{\prime })=1.$$ (18)

Similarly, on a deletion in the descendent sequence ($a_{0,k}^{\prime }\neq "-"\;\mathrm{and}\;b_{0,k}^{\prime }="-"$), we have:

$$P(a_{h,k}^{\prime }|a_{0,k}^{\prime },b_{0,k}^{\prime })=\pi_{a_{h,k}^{\prime }}^{h},$$ (19)

$$P({b_{h,k}^{\prime }|a}_{h,k}^{\prime },a_{0,k}^{\prime },b_{0,k}^{\prime })=1.$$ (20)

Altogether, $P(a_{h,k}^{\prime }|a_{0,k}^{\prime },b_{0,k}^{\prime })$ and $P(b_{h,k}^{\prime }|a_{h,k}^{\prime },a_{0,k}^{\prime },b_{0,k}^{\prime })$ are given by:

$$P({b_{h,k}^{\prime }|a}_{h,k}^{\prime },a_{0,k}^{\prime },b_{0,k}^{\prime })=\{\begin{array}{cc} g_{a_{h,k}^{\prime }{,b}_{h,k}^{\prime }}(t), & a_{0,k}^{\prime },b_{0,k}^{\prime }\neq "-"\\ \pi_{b_{h,k}^{\prime }}^{h}, & a_{0,k}^{\prime }="-"\;and\;b_{0,k}^{\prime }\neq "-"\\ 1, & a_{0,k}^{\prime }\neq "-"\;and\;b_{0,k}^{\prime }="-" \end{array},$$ (21)

$$P(a_{h,k}^{\prime }|a_{0,k}^{\prime },b_{0,k}^{\prime })=\{\begin{array}{cc} \pi_{a_{h,k}^{\prime }}^{h}, & a_{0,k}^{\prime }\neq "-"\\ 1, & a_{0,k}^{\prime }="-"\;and\;b_{0,k}^{\prime }\neq "-" \end{array}.$$ (22)

#### Model B

Model B assumes that epigenomic changes depend on both sequence substitutions and indels (Model B, Fig 2). Similar to Model M, the epigenomic change is modeled with the Poisson process $g_{a_{h,k}^{\prime }{,b}_{h,k}^{\prime }}(t)$ on a matched base ($a_{0,k}^{\prime }=b_{0,k}^{\prime }$), whereas on a position with substitution ($a_{0,k}^{\prime }\neq b_{0,k}^{\prime }$ and $a_{0,k}^{\prime },b_{0,k}^{\prime }\neq "-"$), the descendent epigenomic state is modeled by the equilibrium probabilities. On indels ($a_{0,k}^{\prime }="-"\;\mathrm{or}\;b_{0,k}^{\prime }="-"$), the epigenomic state is modeled using the equilibrium probability following Eqs (17)–(20).

$$P(b_{h,k}^{\prime }|a_{h,k}^{\prime },a_{0,k}^{\prime },b_{0,k}^{\prime })=\{\begin{array}{cc} g_{a_{h,k}^{\prime }{,b}_{h,k}^{\prime }}(t), & a_{0,k}^{\prime }=b_{0,k}^{\prime }\\ \pi_{b_{h,k}^{\prime }}^{h} & a_{0,k}^{\prime }\neq b_{0,k}^{\prime }\;and\;b_{0,k}^{\prime }\neq "-"\\ 1, & a_{0,k}^{\prime }\neq "-"\;and\;b_{0,k}^{\prime }="-" \end{array},$$ (23)

$$P(a_{h,k}^{\prime }|a_{0,k}^{\prime },b_{0,k}^{\prime })=\{\begin{array}{cc} \pi_{a_{h,k}^{\prime }}^{h}, & a_{0,k}^{\prime }\neq "-"\\ 1 & a_{0,k}^{\prime }="-"\;and\;b_{0,k}^{\prime }\neq "-" \end{array}.$$ (24)

### A unified evolutionary model incorporating all four hypotheses

We express the probability of two homologous genomic regions as a mixture of the four models, thus obtained a general probabilistic model that do not depend on any of the specific hypothesis as follows:

P(A,B)=P(A,B|M)P(M)+P(A,B|N)P(N)+P(A,B|I)P(I)+P(A,B|B)P(B), (25)

where *P*(*A*,*B*|*M*), *P*(*A*,*B*|*N*), *P*(*A*,*B*|*I*), and *P*(*A*,*B*|*B*) are the four probability density functions for Models M, N, I, and B, respectively.

### Dynamic programming algorithms

#### Model I and Model B

The dynamic programming algorithm was built on top of the parameter estimation algorithm for the TKF model [4]. We implemented a simplification of the procedure [52], which has been shown to vastly reduce the runtime of the algorithm. In the algorithm, the computation of the probabilities of sequence changes was adopted from the TKF model. The computation of the probabilities of epigenomic modifications was integrated into the recursive procedure by including (1) the products of equilibrium probabilities of epigenomic modification states, namely $\prod_{h=1}^{H}\pi_{b_{h,i}}^{h}$; (2) the changes of epigenomic modification states on matches and mismatches, namely $\prod_{h=1}^{H}G(a_{h,m},b_{h,n},a_{0,m},b_{0,n})$.

Since *P*(*A*,*B*) = *P*(*A*)*P*(*B*|*A*), and *P*(*A*) can be directly computed, only the computation of *P* (*B*|*A*) requires dynamic programming. Denote the (*s*_*A*_ + 1) × (*s*_*B*_ + 1) matrix in the dynamic programming algorithm by *L*, *L* was computed following rules listed below.

Boundary conditions

$$L_{0,0}=p_{1}^{\prime \prime }(t)$$ (26)

$$L_{m,0}=p_{1}^{\prime \prime }(t){p_{0}^{\prime }(t)}^{m}$$ (27)

$$L_{0,n}=p_{n+1}^{\prime \prime }(t)\prod_{i=1}^{n}(\pi_{b_{0,i}}\prod_{h=1}^{H}\pi_{b_{h,i}}^{h})$$ (28)

Recursive procedure

$$\begin{array}{l} L_{m,n}=p_{0}^{\prime }(t)L_{m-1,n}+{\lambda \beta \pi }_{b_{0,n}}\left(\prod_{h=1}^{H}\pi_{b_{h,n}}^{h}\right)L_{m,n-1}\\ \;+[f_{a_{0,m},b_{0,n}}(t)p_{1}(t)\prod_{h=1}^{H}G(a_{h,m},b_{h,n},a_{0,m},b_{0,n})\\ \;+(p_{1}^{\prime }(t)-\lambda \beta p_{0}^{\prime }(t))\pi_{b_{0,n}}\prod_{h=1}^{H}\pi_{b_{h,n}}^{h}]L_{m-1,n-1} \end{array}$$ (29)

All of the functions *p*,*p*′,*p*″ were given by the TKF model [4]. For Model I, the term *G*(*a*_*h*,*m*_,*b*_*h*,*n*_,*a*_0,*m*_,*b*_0,*n*_) was $g_{a_{h,m},b_{h,n}}(t)$. For Model B, the term was the same as for Model I on matched bases, while it became $\pi_{b_{h,n}}^{h}$ on mismatches.

#### Model N and Model M

The dynamic programming algorithm was implemented using a similar idea as for Model I and B, except that the computation of *P*(*A*^*h*^) became part of the recursive procedure, and the function *G* changed. Given Eqs (8) and (10):

$$\Phi_{B}(a_{h,k}^{\prime })=\varphi_{B0}^{h}g_{0,a_{h,k}^{\prime }}(t)+\varphi_{B1}^{h}g_{1,a_{h,k}^{\prime }}(t)$$

$$\Phi_{A}(b_{h,k}^{\prime })=\varphi_{A0}^{h}g_{0,b_{h,k}^{\prime }}(t)+\varphi_{A1}^{h}g_{1,b_{h,k}^{\prime }}(t)$$

The matrix *L* was computed following rules listed below.

Boundary conditions

$$L_{0,0}=p_{1}^{\prime \prime }(t)$$ (30)

$$L_{m,0}=p_{1}^{\prime \prime }(t){p_{0}^{\prime }(t)}^{m}\prod_{h=1}^{H}\Phi_{B}(a_{h,0})$$ (31)

$$L_{0,n}=p_{n+1}^{\prime \prime }(t)\prod_{i=1}^{n}\left(\pi_{b_{0,i}}\prod_{h=1}^{H}\Phi_{A}(b_{h,0})\right)$$ (32)

Recursive procedure

$$\begin{array}{l} L_{m,n}=p_{0}^{\prime }(t)\left(\prod_{h=1}^{H}\Phi_{B}(a_{h,m})\right)L_{m-1,n}+{\lambda \beta \pi }_{b_{0,n}}\left(\prod_{h=1}^{H}\Phi_{A}(b_{h,n})\right)L_{m,n-1}\\ \;+[f_{a_{0,m},b_{0,n}}(t)p_{1}(t)\prod_{h=1}^{H}\pi_{a_{h,m}}^{h}g_{a_{h,m},b_{h,n}}(t)\\ \;+(p_{1}^{\prime }(t)-\lambda \beta p_{0}^{\prime }(t))\pi_{b_{0,n}}\prod_{h=1}^{H}\Phi_{A}(b_{h,n})\Phi_{B}(a_{h,m})]L_{m-1,n-1} \end{array}$$ (33)

The term *G*(*a*_*h*,*m*_,*b*_*h*,*n*_,*a*_0,*m*_,*b*_0,*n*_) was $g_{a_{h,m},b_{h,n}}(t)$ for Model N (same as Model I). The term for Model M was the same as for Model N on matched bases, while it became $\pi_{b_{h,n}}^{h}$ on mismatches (same as Model B).

### Maximum likelihood estimates

The MLE of *π* and *φ* are calculated by frequency estimates. Due to the relationship $\lambda =\frac{\mu \times (s_{A}+s_{B})}{s_{A}+s_{B}+2}$ [4], the MLE of *λ* is determined as long as the MLE of *μ* is determined. Evolutionary time *t* is set to 1. The remaining parameters *μ*, *s*, and *κ*, collectively denoted as *θ*, are obtained by minimizing the negative log-likelihood function,

l(θ|A,B)=−lnp(A,B|θ). (34)

We used simplex downhill algorithm for this optimization. In each iteration, a number of new parameter sets *θ*^new^ were generated. The algorithm iteratively evaluated each *l*(*θ*^new^|*A*,*B*) and compared it with *l*(*θ*^old^|*A*,*B*) to select the best one, until the minimum was achieved.

### EM algorithm

We proposed an EM algorithm for the maximization of Eq (25),. The algorithm iteratively updates the prior probability of the four models *P*(*N*),*P*(*I*),*P*(*M*),*P*(*B*), as well as the model parameters *μ*, *s*, and *κ*, collectively denoted as *θ*. Let *N* denote the number of region pairs. Let *α*_*K*_ denote the prior probability of the model *K*, where *K* = {*N*,*I*,*M*,*B*}. The algorithm starts from some initial guesses ${\hat{\alpha }}_{K}$ and $\hat{\theta }$ and iterates the following steps until convergence:

**E-step:** update the prior probabilities:

$${\hat{\gamma }}_{\iota K}=\frac{{\hat{\alpha }}_{K}P_{K}(A^{\left(i\right)},B^{\left(i\right)}|\hat{\theta })}{\sum_{K}{\hat{\alpha }}_{K}P_{K}(A^{\left(i\right)},B^{\left(i\right)}|\hat{\theta })}$$ (35)

$${\hat{\alpha }}_{K}^{\mathrm{new}}=\frac{\sum_{i=1}^{N}{\hat{\gamma }}_{\iota K}}{N}$$ (36)

**Μ-step:** update the model parameters and prior probabilities by maximizing the log-likelihood function with the prior probabilities ${\hat{\alpha }}_{K}$:

$${\hat{\theta }}^{\mathrm{new}}=\underset{\theta }{\mathrm{argmax}}\sum_{i=1}^{N}\ln\left\{\sum_{K}{\hat{\alpha }}_{K}P_{K}(A^{\left(i\right)},B^{\left(i\right)}|\hat{\theta })\right\}$$ (37)

### Generating simulation datasets

In the simulation test, we used only one histone modification. The equilibrium probabilities were set to *π*_*A*_ =*π*_*C*_ =*π*_*G*_ =*π*_*T*_ =0.25, *π*_0_ = 0.9,*π*_1_ = 0.1. The simulation data contains 100 500-base-long sequence pairs.

#### Model I and Model B

The simulation data was generated based on the corresponding hypotheses of models. The ancestor was first generated by drawing bases and histone modification states randomly based on the equilibrium probabilities. The alignment path was then generated based on the TKF model. On insertions, the bases and epigenomic modification states were drawn based on the equilibrium probabilities. On matches, the bases and histone modification states were determined based on the substitution probabilities. On mismatches, the bases and histone modification states were also determined based on the substitution probabilities for Model I, while they were drawn based on the equilibrium probabilities for Model B.

#### Model N and Model M

For each region pair, the ancestral sequence was first generated by drawing bases randomly based on the equilibrium probabilities. Local equilibrium probabilities $\varphi_{0}^{h},\varphi_{1}^{h}$ were drawn from a beta distribution $B(1.5,\frac{1.5(1-\pi_{1})}{\pi_{1}})$ and assigned to the ancestral region. After that, the path was determined based on the TKF model. On insertions, the descendent nucleotides were drawn based on the equilibrium probabilities, and the histone modification states were drawn based on the local equilibrium probabilities. On matches, the bases and histone modification states were determined based on the substitution probabilities. On mismatches, the bases and histone modification states were also determined based on the substitution probabilities for Model N, while they were drawn based on the equilibrium probabilities for Model M.

### ChIP-Seq data analyses

#### ChIP-Seq data pre-processing

ChIP-Seq datasets were mapped to human genome assembly hg38, rhesus macaque genome assembly rheMac8 and mouse genome assembly mm10 using bowtie2 with default settings. Duplicated reads and reads with MAPQ<6 were then removed from the data. Peaks were identified using MACS2 with the “broadpeak” option [53].

#### Generating candidate regions for parameter estimation

Peak regions identified in rhesus and mouse RS H3K4me3 ChIP-Seq data were first remapped to human genome using liftOver with minMatch = 0.5 [54]. For each pair of species (human vs. rhesus and human vs. mouse), three files were merged: (1) The DNase I hypersensitivity peak clusters derived from 95 human cell lines; (2) H4K3me3 peaks identified in the human RS H3K4me3 ChIP-Seq data; (3) remapped H3K4me3 peaks identified in the rhesus or mouse RS H3K4me3 ChIP-Seq data.

Human-rhesus merged regions were trimmed to no longer than 500bp, and remapped to rhesus macaque genome using liftOver with minMatch = 0.5 to find their homologous regions. The H3K4me3 ChIP-Seq data was distributed to these region pairs based on the identified peak regions. 8,000 regions were randomly sampled for parameter estimation. The four models were fitted separately using the MLE algorithm. Human-mouse homologous regions were identified by remapping merged regions to mouse genome directly using the liftOver chain file with single gaps up to 10bp. 10,000 region pairs were randomly sampled for parameter estimation. The models were fitted using the EM algorithm.

For region classification, only human and rhesus/mouse H3K4me3 peaks were merged. The merged regions were trimmed to no longer than 2,000bp, and remapped to rhesus genome using liftOver with minMatch = 0.1. Remapped regions with less than 90% realigned successfully were extended to the length of the original ones. The H3K4me3 ChIP-Seq data was then distributed to these region pairs based on the identified peak regions.

#### Homologous region classification

For model comparison and homologous region classification, regions with H3K4me3 in either species were merged, trimmed, and remapped to the other species to find their homologous regions. For each homologous pair, four likelihoods were obtained using the four models. Each homologous pair was categorized to one model by the largest likelihood.

#### Separating evolutionary impacts of sequence substitutions and indels

We leveraged the four models to evaluate the relative impacts on interspecies epigenomic variations from substitutions and that from indels. By integrating out substitution’s impacts from the probability models, we obtained the overall impacts of indels, and vice versa. We introduce the binary variable *mut* to indicate independence *mut* = 0 and dependence *mut* = 1 to substitutions, and binary variable *ind* to indicate independence of dependence to indels. The probability of observing a region pair given *mut* or *ind* can be expressed as a combination of likelihoods yielded by different models:

$$P(A,B|mut=0)=\sum_{ind}P(A,B,ind|mut=0)=\sum_{ind}P(A,B|ind,mut=0)P(ind)$$ (38)

$$P(A,B|mut=0)=aP_{N}(A,B)+(1-a)P_{I}(A,B)$$ (39)

The coefficient *a* was estimated using the frequency of regions within which epigenomic changes are independent to indels. Similarly, we also have

$$P(A,B|mut=1)=aP_{M}(A,B)+(1-a)P_{B}(A,B)$$ (40)

$$P(A,B|ind=0)=bP_{N}(A,B)+(1-b)P_{M}(A,B)$$ (41)

$$P(A,B|ind=1)=bP_{I}(A,B)+(1-b)P_{B}(A,B)$$ (42)

The coefficient *b* was estimated using the frequency of regions within which epigenomic changes are independent to substitutions.

We proposed two normalized likelihood ratios, *r*_*ind*_ and *r*_*mut*_ to assess the effect of indels and substitutions:

$$r_{ind}=\frac{l(ind=1)-l(ind=0)}{\left|l(ind=1)+l(ind=0)\right|},\;r_{mut}=\frac{l(mut=1)-l(mut=0)}{\left|l(mut=1)+l(mut=0)\right|}$$ (43)

where *l*(*ind*) = log *P*(*A*,*B*|*ind*), *l*(*mut*) = log *P* (*A*,*B*|*mut*). In Eq (43), the likelihood differences were normalized to the absolute values of the sums of the likelihoods in order to eliminate the effects of various region lengths. *r*_*ind*_ and *r*_*mut*_ represented the dependency of epigenomic changes on indels and substitutions. The extent of independence increased as the ratios increased.

An alternative normalization approach was also evaluated, where the likelihood differences were normalized to the region pair lengths, which was defined as the sum of both regions in a pair:

$$r_{ind}=\frac{l(ind=1)-l(ind=0)}{s_{A}+s_{B}},\;r_{mut}=\frac{l(mut=1)-l(mut=0)}{s_{A}+s_{B}}$$ (44)

The two approaches were compared to assess if the results were sensitive to different normalization methods.

#### Searching for species-specific transposon insertions

We selected local-sequence-independent regions with the top 10%, 5% and 2% *r*_*ind*_ values and local-sequence-dependent regions with the top 10%, 5% and 2% |*r*_*ind*_| values for transposon analyses. We downloaded RepeatMasker files for corresponding genome references as transposon annotations. Transposons that could be remapped to the other species were removed to exclude the ones shared by the two species. For each region pair, transposons covered 50% of the region or with 50% covered by the region were kept. Transposons longer than 500bp within the two regions were compared, and the ones with identical family, class and name were removed to keep species-specific transposon insertions. Regions with species-specific transposon insertions were kept and classified into three categories: conserved peak regions, transposon-induced peak regions and transposon-disrupted peak regions. Families and classes of species-specific transposon insertions were then summarized in each category. Here we designated gains and losses to reflect the relationship between species-specific peak regions and insertions in the sequence. A species-specific epigenomic peaks was designated as “epigenomic gain” if it overlapped with a transposon insertion in the same species, or “epigenomic loss” if the transposon insertion occurred in the other species.

Odds ratios were used to measure the enrichment levels of different transposon families within transposon-induced and transposon-repressed peaks. For transposon-induced peaks, region pairs with transposon-involved peaks were selected as the union set. For each transposon family, a contingency table was built to reflect whether the particular type of transposon overlapped with a transposon-induced peak. For transposon-disrupted peaks, region pairs within which transposon insertions occurred at the opposite side of peaks were selected as the union set, and the contingency table was built based on whether the specific type of transposon insertion occurred together with a peak removal. Odds ratios were computed based on the contingency tables, and the significance levels were assessed using 95% confidence intervals and chi-square tests.

#### Motif analysis

Sequence motifs were identified within the LTR-ERV1 transposons found in the transposon-induced peaks with *r*_*ind*_ > 0.01 (target transposons). All LTR transposons in the ERV1 family longer than 500bp were used as background (background transposons). The *de novo* motif discovery was performed using Homer [52] with default parameters.

## Supporting information

S1 Fig Parameter deviation values and log likelihood values vs. iteration. (A) The logarithms of the ratios of estimated parameters (***s***,***μ***,***κ***) to the true values (s*,μ*,κ*) vs. iteration number. All estimated parameters converged to the true values (logarithms of the ratio equaled to zero) over iterations. (B) The logarithms of the ratios of true likelihood values to calculated likelihood values vs. iteration number. This showed that all negative logarithms of calculated likelihood decreased monotonically over iterations to a value close to the true, meaning that the optimization algorithm was working as intended.
(PDF)

S2 Fig Percent error of estimated parameters. Each panel shows the percent error of parameters for one specific model. In one panel, every column represents 10 simulation tests under one simulation condition (as shown in Table S1). The estimated parameter of which the percentage error is plotted is labeled below the column. The color of the column shows the true value of the estimated parameter.
(PDF)

S3 Fig Comparison of negative logarithms of likelihood with different models upon datasets based on different hypotheses. Each column corresponds to a simulation dataset. Models corresponding to the hypotheses of the datasets showed highest logarithm of likelihood, showing that those models fit the corresponding datasets best.
(PDF)

S4 Fig An example of both types of sequence changes (substitution and indel) contributing to the independency of epigenetic variation. Within the regions shown in the figure, substitutions and indels both contributed to sequence changes between the two homologous sequences, whereas the H3K4me3 peak remained conserved. Neither substitution nor indel appeared to relate to interspecies variation of the peak.
(PDF)

S5 Fig Scatterplot of all homologous regions showing the degree of dependence to indels (x-axis) versus the degree of dependence to substitutions (y-axis). The two metrics ***r***_***ind***_ and ***r***_***mut***_ were derived following Eq (43), in which the likelihood differences were normalized by region pair lengths.
(PDF)

S6 Fig Counts of class and family of transposons among different types of peak regions with the largest 5% and 2% |*r*_*ind*_|. The number of class and family of transposons among different types of peaks (up to bottom): conserved peaks; transposon-induced peaks; transposon-disrupted peaks.
(PDF)

S7 Fig Relative levels of enrichment of each transposon type within transposon-induced and transposon-disrupted peaks between human and rhesus. Log odds ratio > 0 or < 0 corresponds to an increased or decreased level of enrichment. Error bars represent 95% confidence interval of log odds ratios. *: p-value of chi-square test < 0.05. **: p-value of chi-square test < 0.01. ***: p-value of chi-square test < 0.001.
(PDF)

S8 Fig H3K4me3 ChIP-Seq intensities within the LINE-L1-disrupted H3K4me3 peaks with L1 insertions masked. (A) Demonstration of a L1-disrupted H3K4me3 peak. Shaded bands between two species indicate homologous sequences. Sequence with hatch pattern: insertion of a LINE-L1 transposon in Species 1, which was excluded in the analyses. (B) Log fold changes of H3K4me3 ChIP-Seq read counts between regions without L1 insertions and their corresponding homologous regions. (C) Log H3K4me3 ChIP-Seq intensities within regions without L1 insertions (left column) and flanking regions of the L1 insertions (right column).
(PDF)

S9 Fig An example of epigenetic variations with sequence changes between human and rhesus. A pair of homologous regions exhibiting indel-associated H3K4me3 loss. The insertion of a LINE-L1 transposon (a) in rhesus macaque between two ERVL-MaLR family repeats (b, c) is associated with the loss of an H3K4me3 peak.
(PDF)

S10 Fig Distance characteristics of regions classified into different models. Panels A, B, E and F show distributions of distance between the region pairs and transcription start sites (TSSs) of their nearest genes, while Panels C, D, G and H show distributions of distance between the region pairs and transcription end sites (TESs) of their nearest genes. Panels A, C, E and G represent the region pairs with local-sequence-independent H3K4me3 variations, while Panels B, D, F and H represent the region pairs with local-sequence-dependent H3K4me3 changes. Panels A through D depict the regions in human while Panels E through H depict the regions in rhesus monkey.
(PDF)

S11 Fig Classifications of human-mouse homologous genomic regions. (A) Proportions of human-mouse homologous regions classified into each model. (B) Scatterplot of all homologous regions showing the degree of dependence to indels (***r***_***ind***_, x axis) versus the degree of dependence to substitutions (***r***_***mut***_, y axis). Actual data for selected homologous regions (red circles) were given in panel (C)—(F). (C-D): Examples of regions with local-sequence-dependent H3K4me3 changes, within which the insertions of LINE-L1 transposons are associated with H3K4me3 loss in mouse and human, respectively. (E-F): Examples of regions with local-sequence-independent H3K4me3 variations. (E): The H3K4me3 variation is independent of both substitutions and indels. (F): The H3K4me3 variation is independent of large sequence insertions.
(PDF)

S12 Fig Enrichment levels of each transposon type within transposon-induced and transposon-disrupted peaks between human and mouse. (A) Counts of different transposon families within different types of peak (B) Odds ratios between different transposon families and transposon-involved peaks. Log odds ratio > 0 or < 0 corresponds to an increased or decreased level of enrichment. Error bars represent 95% confidence interval of log odds ratios. *: p-value of chi-square test < 0.05. **: p-value of chi-square test < 0.01. ***: p-value of chi-square test < 0.001.
(PDF)

S1 Table Parameters used for simulation. A total of 8 datasets (Simulation column) were simulated with corresponding parameters (Columns *s*, *μ*, and *κ*) under each model. Other parameters are given in Methods.
(PDF)

S2 Table Evolutionary parameters estimated from sampled regions. Three randomly sampled subsets of all homologous region pairs were used to estimate parameters for all four models. Parameters remained close to each other among all subsets.
(PDF)

S3 Table Parameters estimated using different MACS2 q-value cutoffs. (PDF)

S4 Table Number of insertion-involved homologous region pairs selected with different *r*_*ind*_ percentage cutoffs. (PDF)
