# Supplementary figures and images for: A likelihood approach to testing hypotheses on the co-evolution of epigenome and genome
Source: PLoS Comput Biol. 2018 Dec 26;14(12):e1006673. doi: 10.1371/journal.pcbi.1006673 (PMC6324829; doi:10.1371/journal.pcbi.1006673)

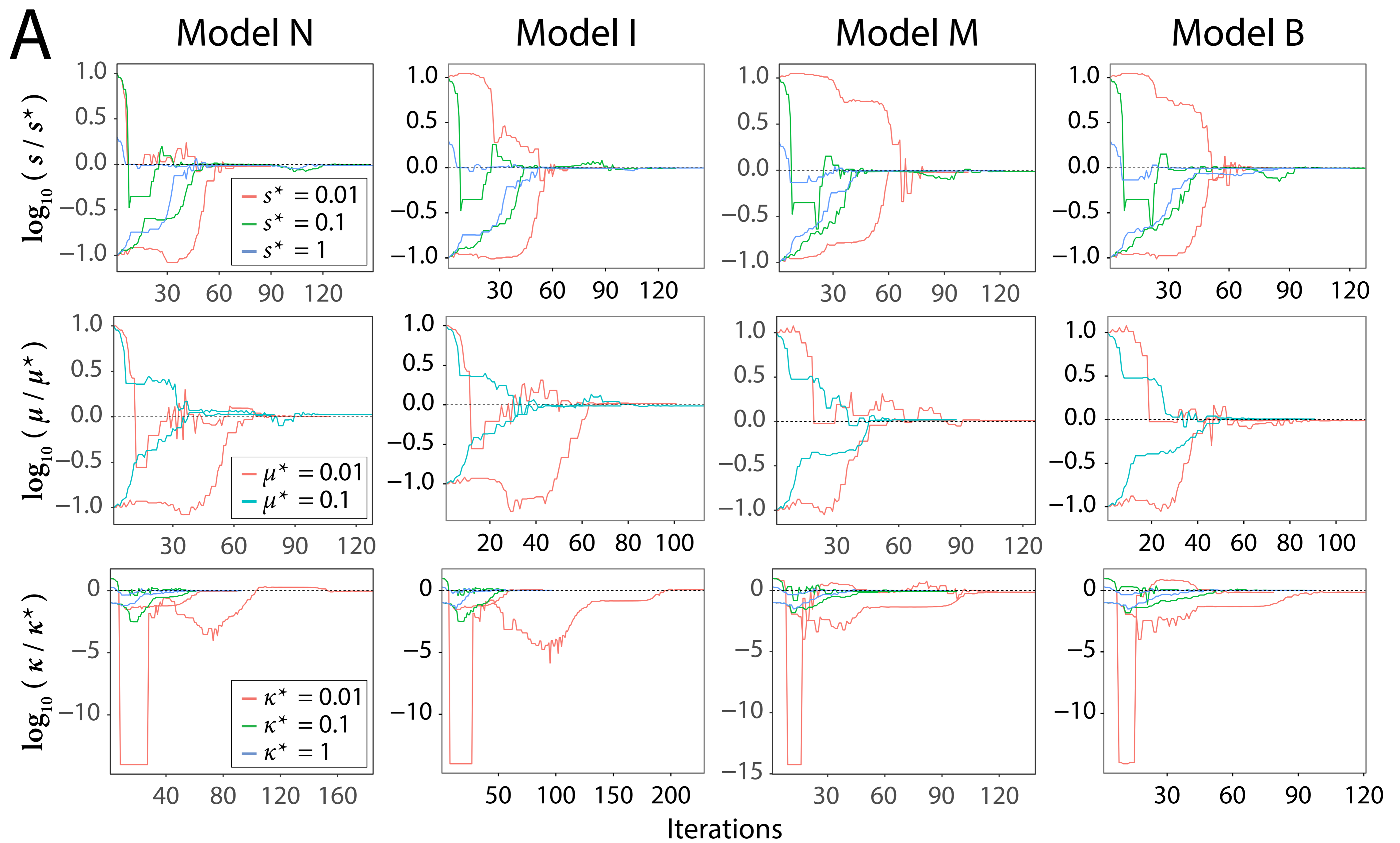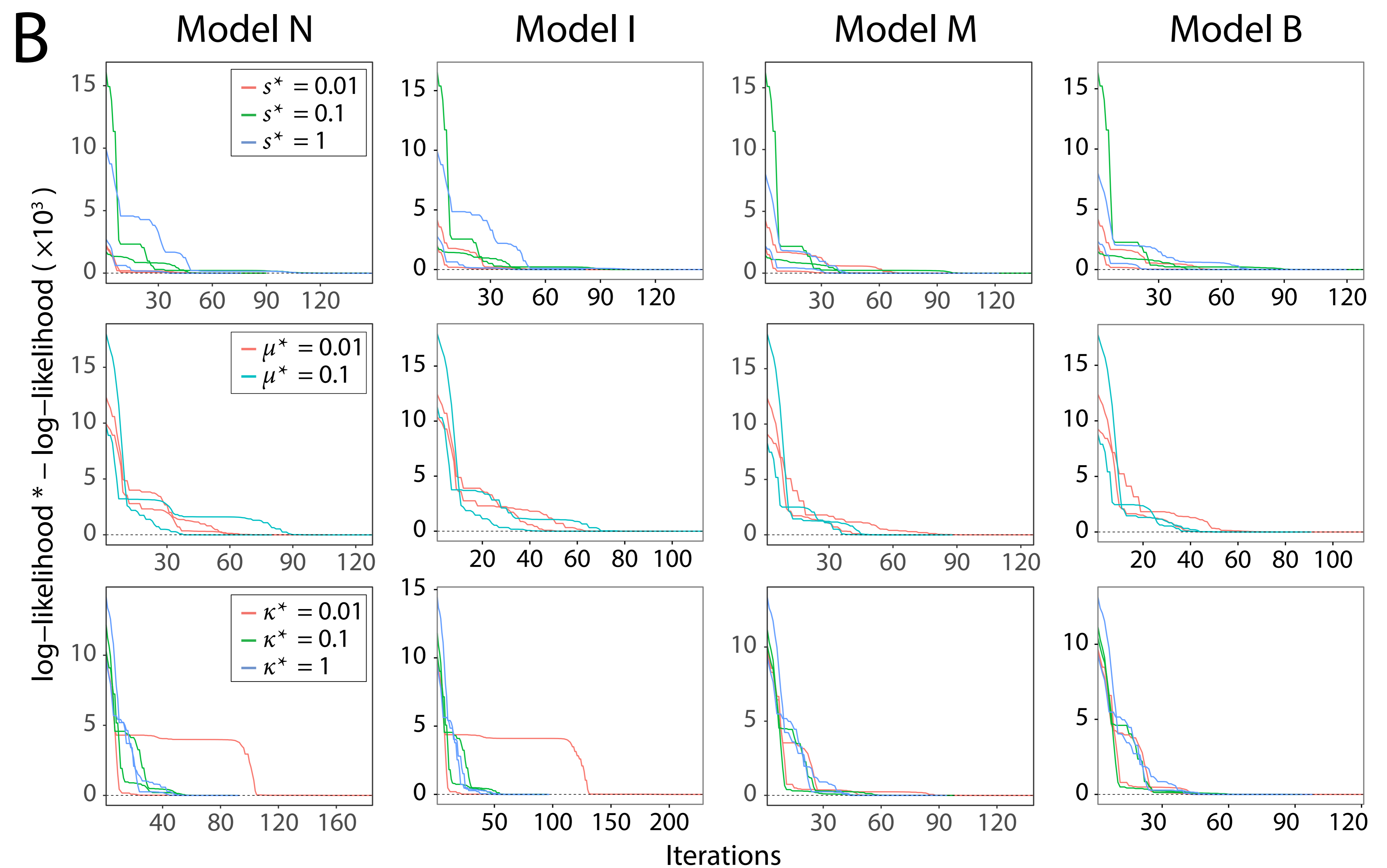

Supplement: S1 Fig — (A) The logarithms of the ratios of estimated parameters (s,μ,κ) to the true values (s*,μ*,κ*) vs. iteration number. All estimated parameters converged to the true values (logarithms of the ratio equaled to zero) over iterations. (B) The logarithms of the ratios of true likelihood values to calculated likelihood values vs. iteration number. This showed that all negative logarithms of calculated likelihood decreased monotonically over iterations to a value close to the true, meaning that the optimization algorithm was working as intended. (PDF) [file pcbi.1006673.s001.pdf]

True value = 0.01   True value = 0.1   True value = 1

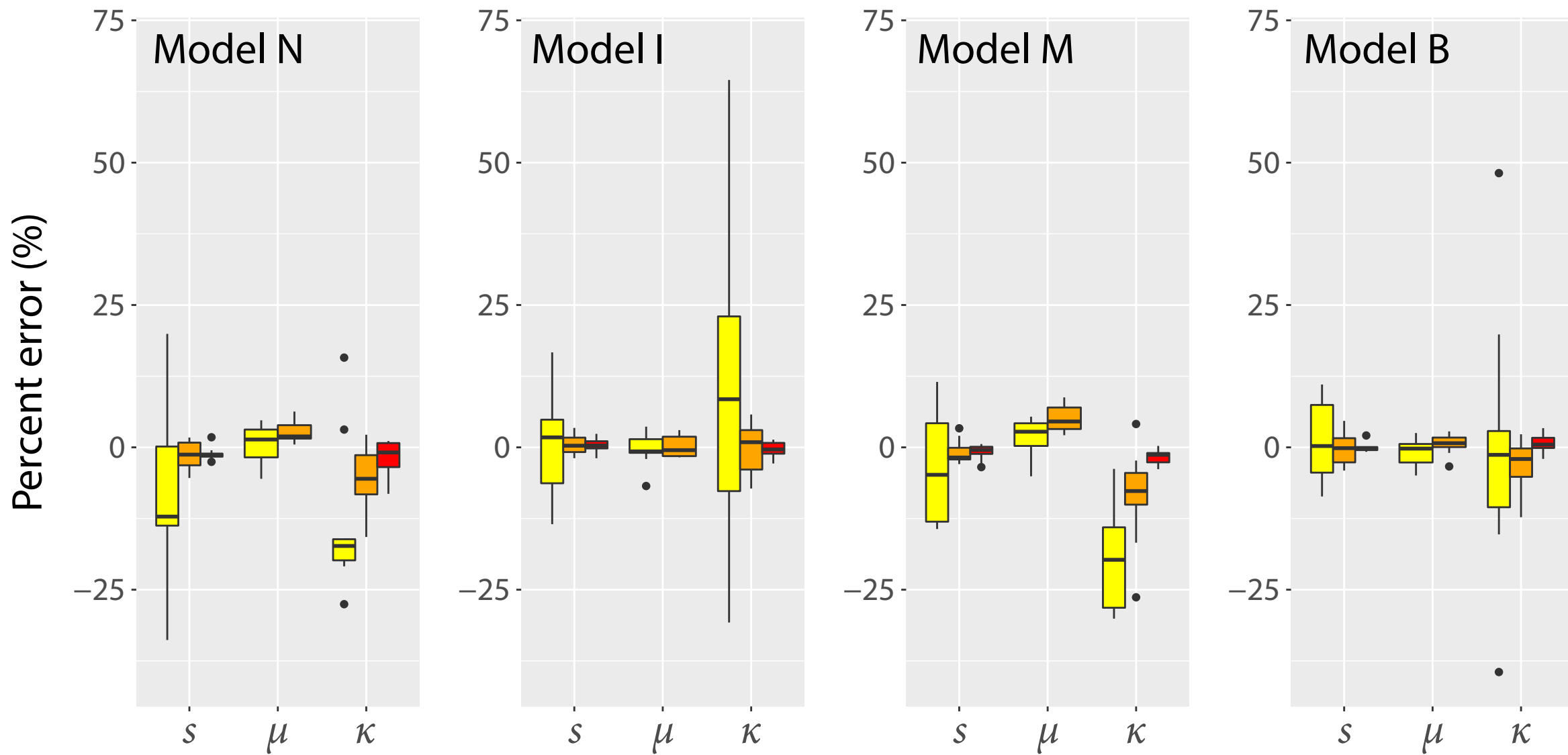

Supplement: S2 Fig — Each panel shows the percent error of parameters for one specific model. In one panel, every column represents 10 simulation tests under one simulation condition (as shown in Table S1). The estimated parameter of which the percentage error is plotted is labeled below the column. The color of the column shows the true value of the estimated parameter. (PDF) [file pcbi.1006673.s002.pdf]

Log-likelihood

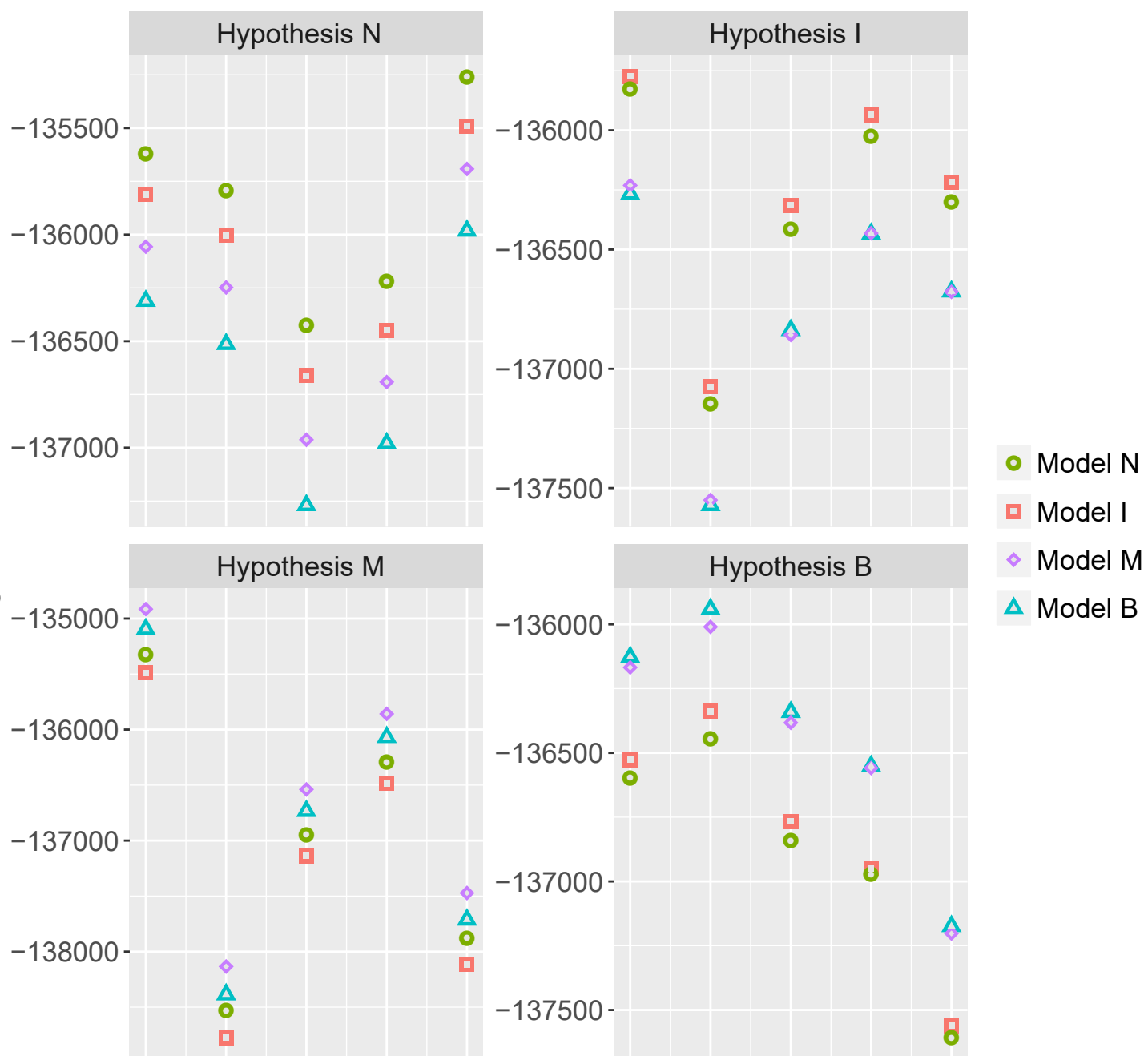

Supplement: S3 Fig — Each column corresponds to a simulation dataset. Models corresponding to the hypotheses of the datasets showed highest logarithm of likelihood, showing that those models fit the corresponding datasets best. (PDF) [file pcbi.1006673.s003.pdf]

# Human

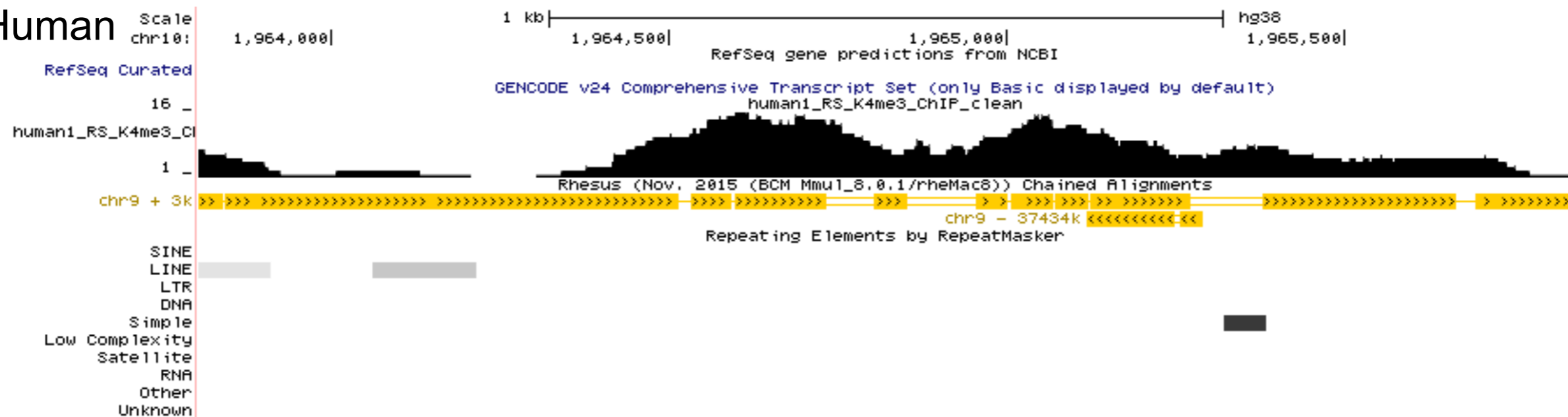

# Rhesus

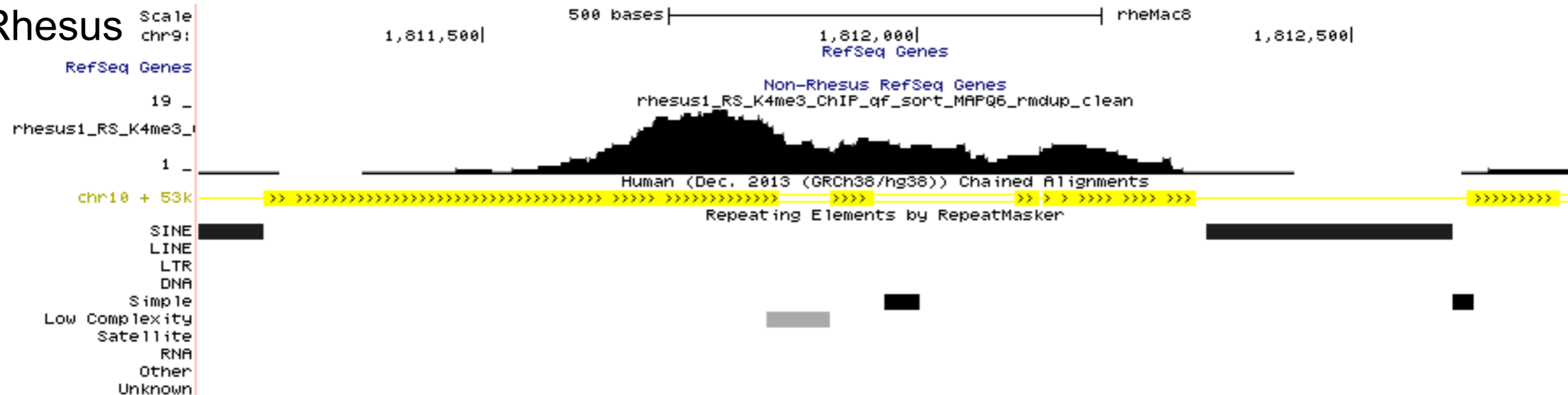

Supplement: S4 Fig — Within the regions shown in the figure, substitutions and indels both contributed to sequence changes between the two homologous sequences, whereas the H3K4me3 peak remained conserved. Neither substitution nor indel appeared to relate to interspecies variation of the peak. (PDF) [file pcbi.1006673.s004.pdf]

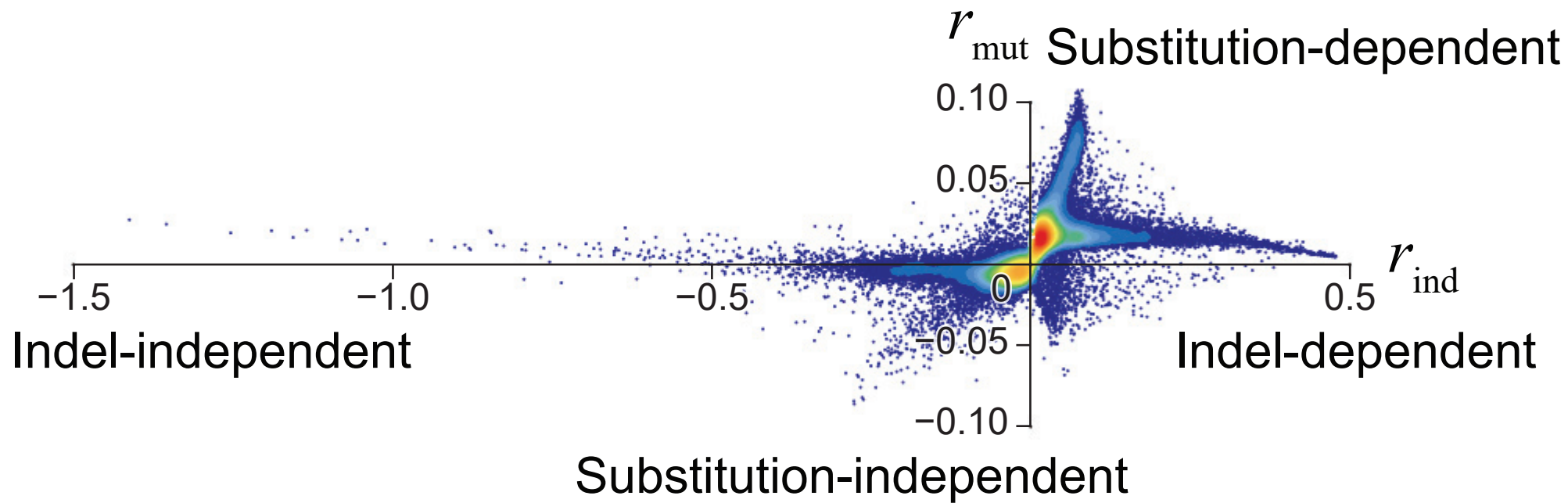

Supplement: S5 Fig — The two metrics rind and rmut were derived following Eq (43), in which the likelihood differences were normalized by region pair lengths. (PDF) [file pcbi.1006673.s005.pdf]

# Top 5%

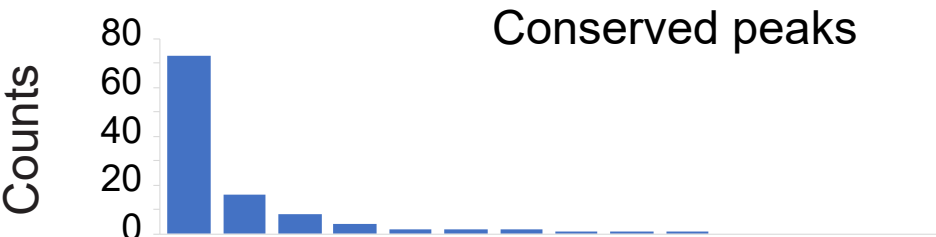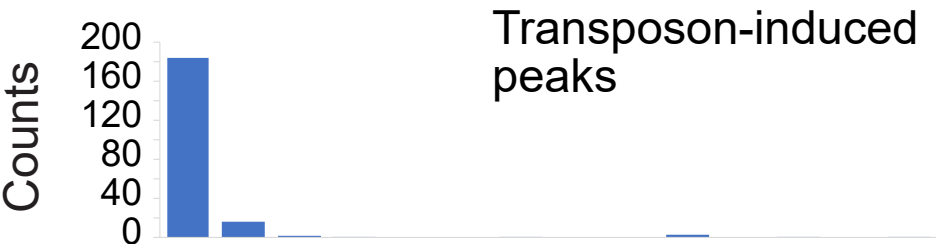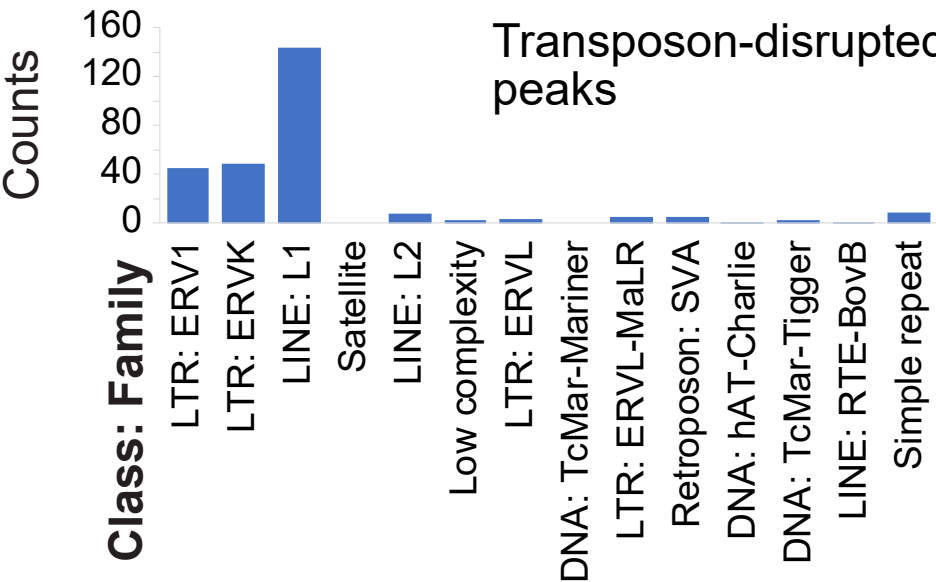

# Top 2%

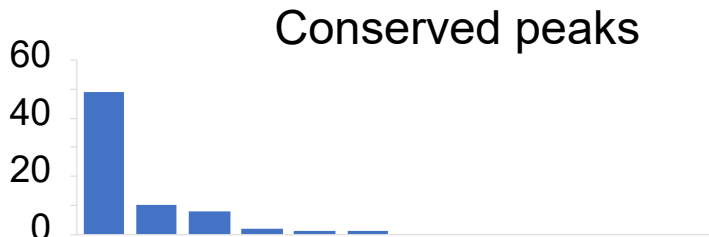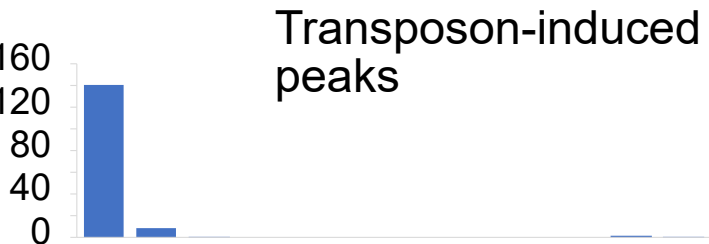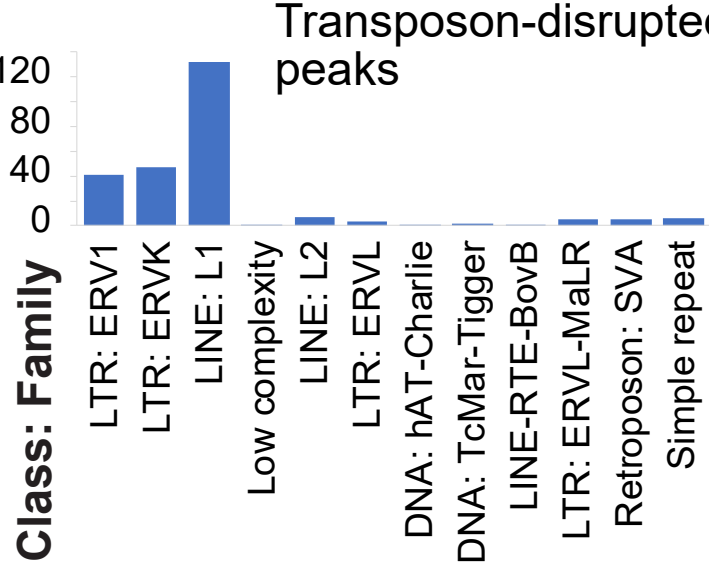

Supplement: S6 Fig — The number of class and family of transposons among different types of peaks (up to bottom): conserved peaks; transposon-induced peaks; transposon-disrupted peaks. (PDF) [file pcbi.1006673.s006.pdf]

# Human

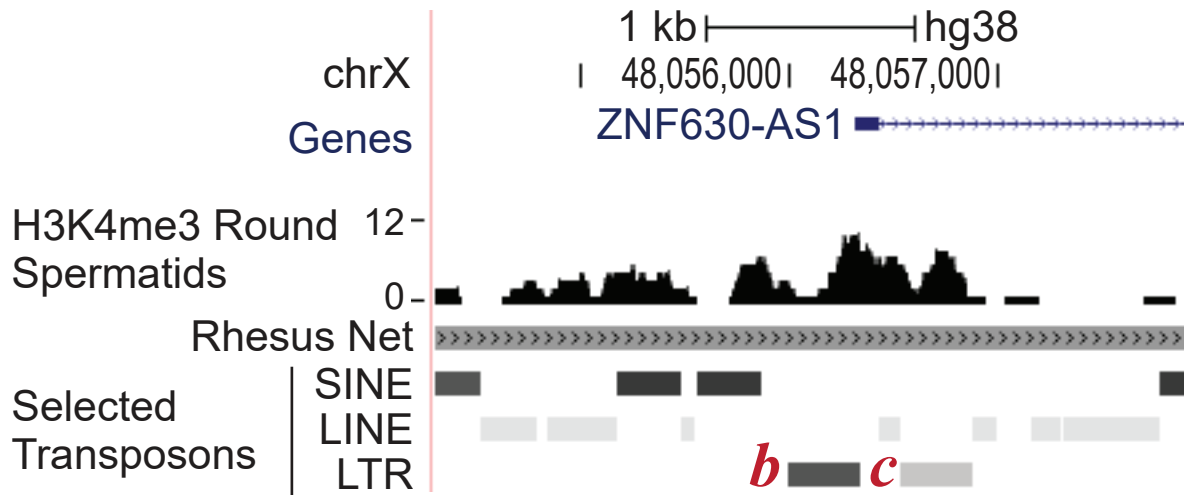

# Rhesus macaque

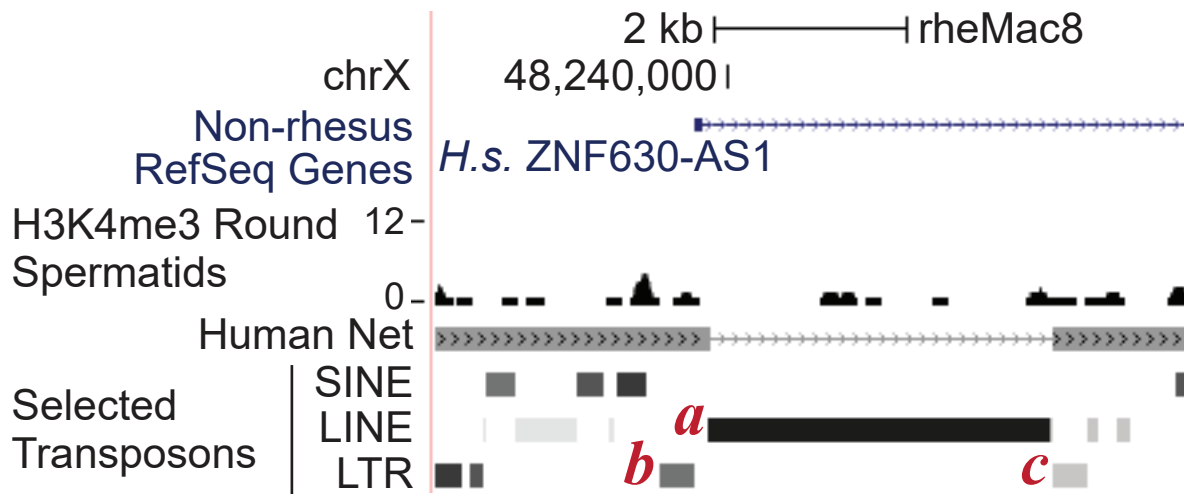

Supplement: S9 Fig — A pair of homologous regions exhibiting indel-associated H3K4me3 loss. The insertion of a LINE-L1 transposon (a) in rhesus macaque between two ERVL-MaLR family repeats (b, c) is associated with the loss of an H3K4me3 peak. (PDF) [file pcbi.1006673.s009.pdf]

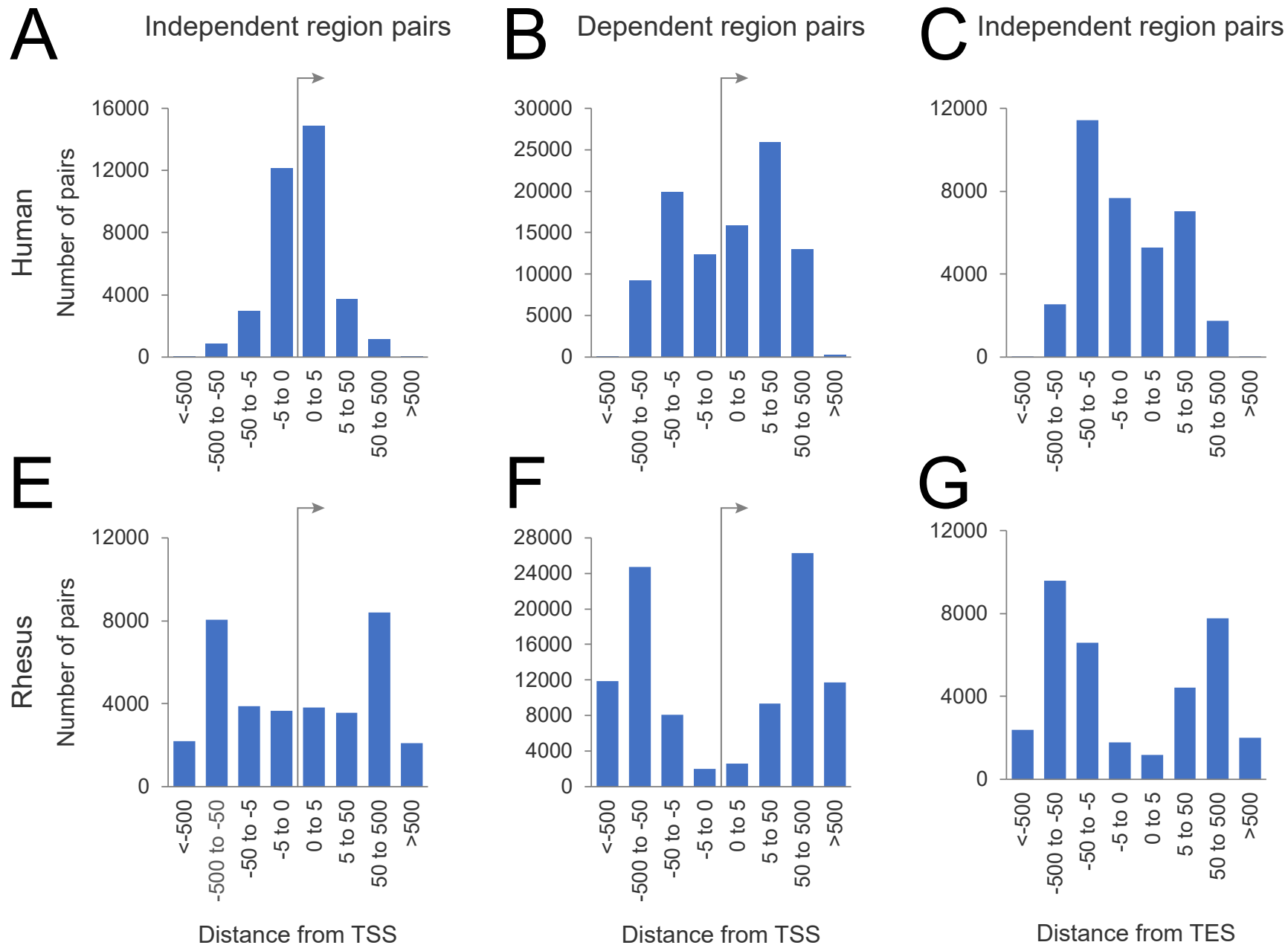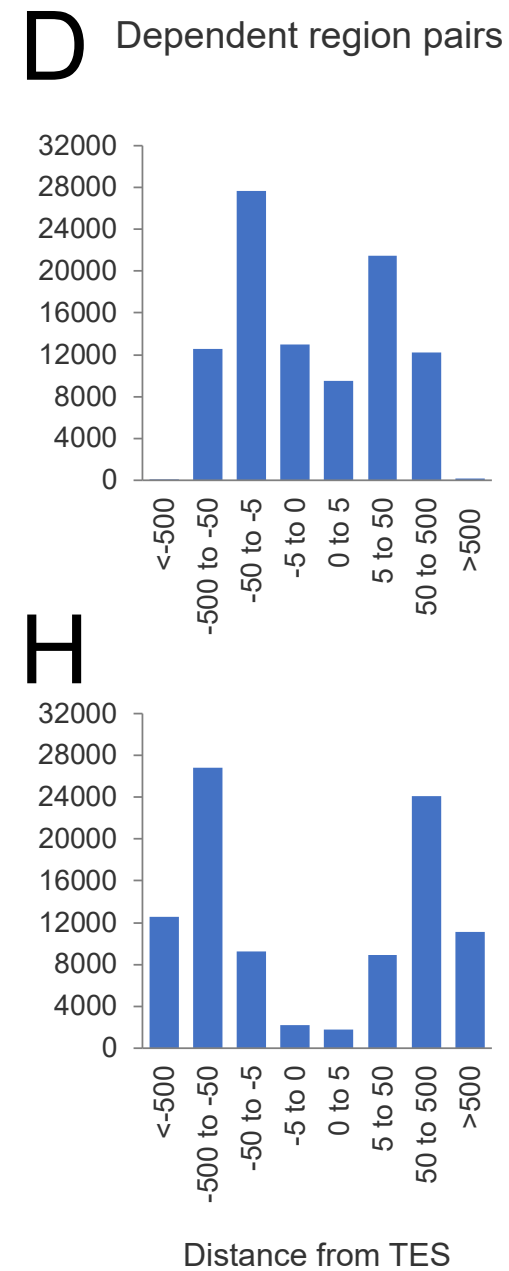

Supplement: S10 Fig — Panels A, B, E and F show distributions of distance between the region pairs and transcription start sites (TSSs) of their nearest genes, while Panels C, D, G and H show distributions of distance between the region pairs and transcription end sites (TESs) of their nearest genes. Panels A, C, E and G represent the region pairs with local-sequence-independent H3K4me3 variations, while Panels B, D, F and H represent the region pairs with local-sequence-dependent H3K4me3 changes. Panels A through D depict the regions in human while Panels E through H depict the regions in rhesus monkey. (PDF) [file pcbi.1006673.s010.pdf]

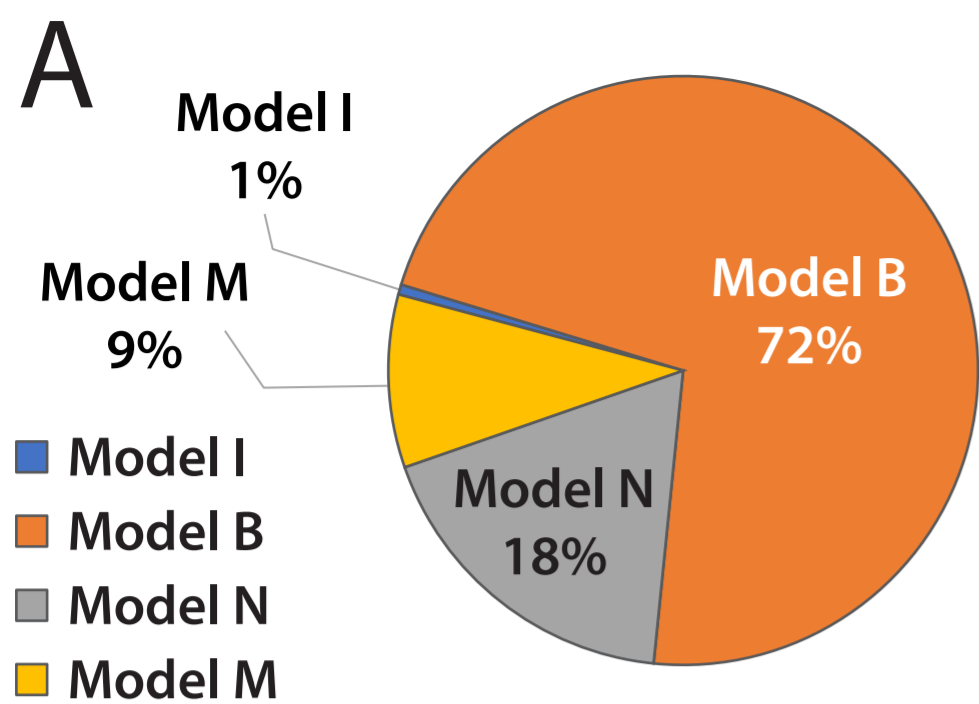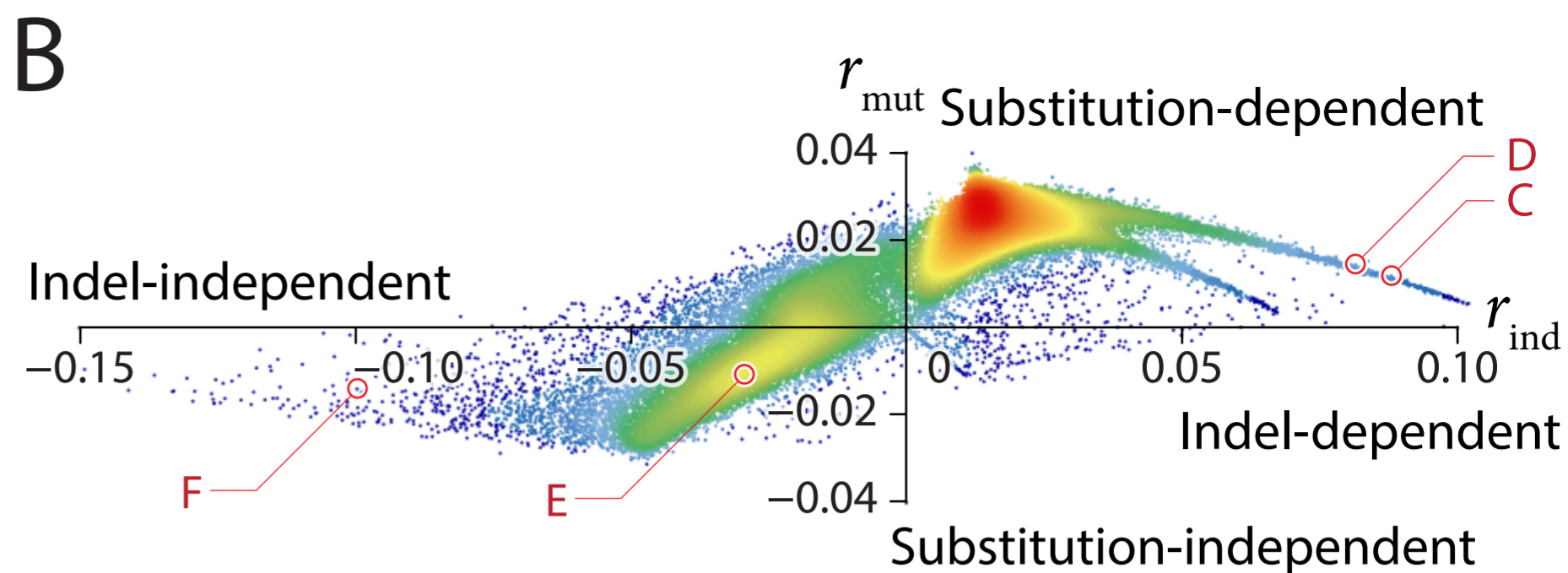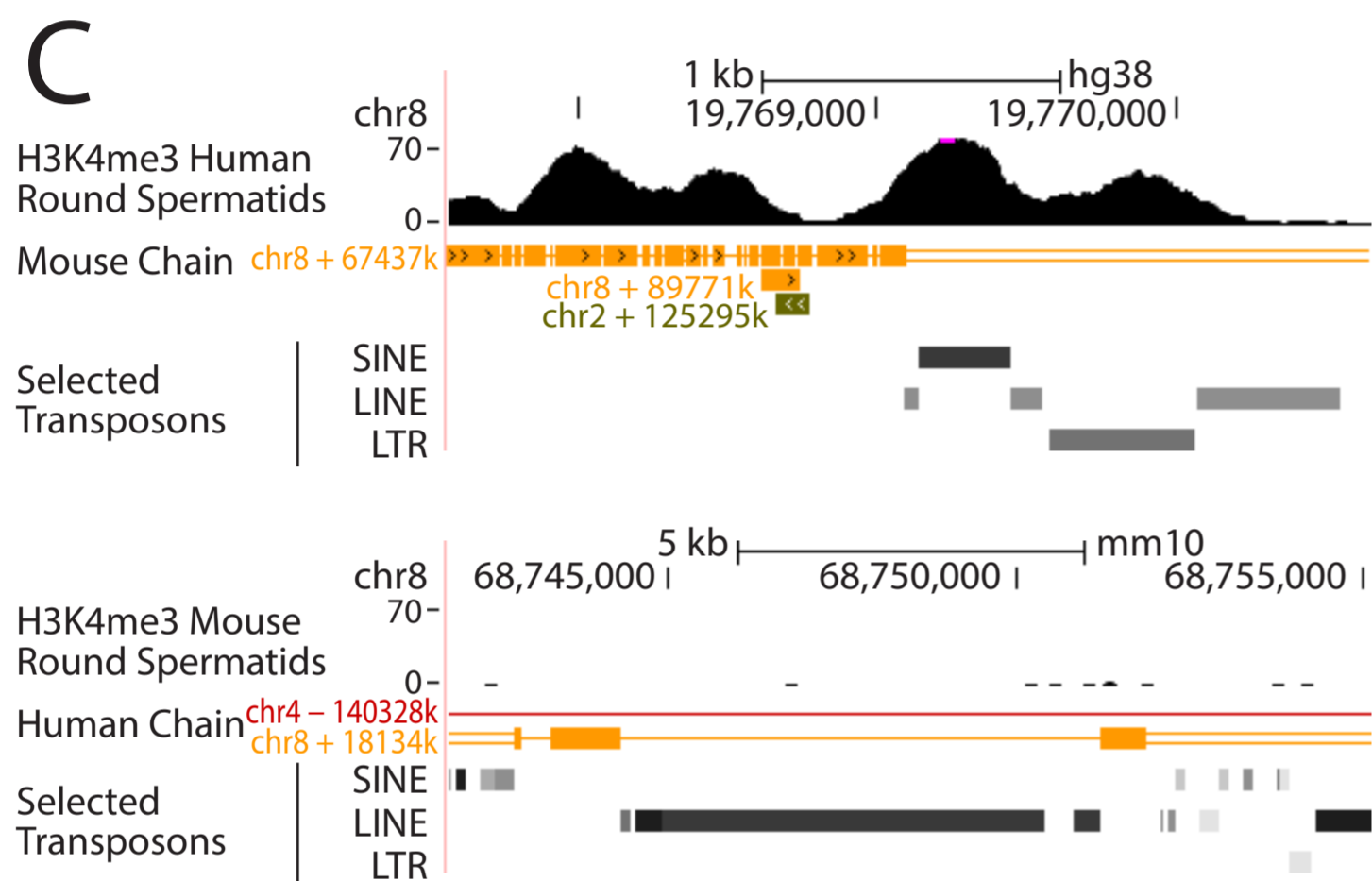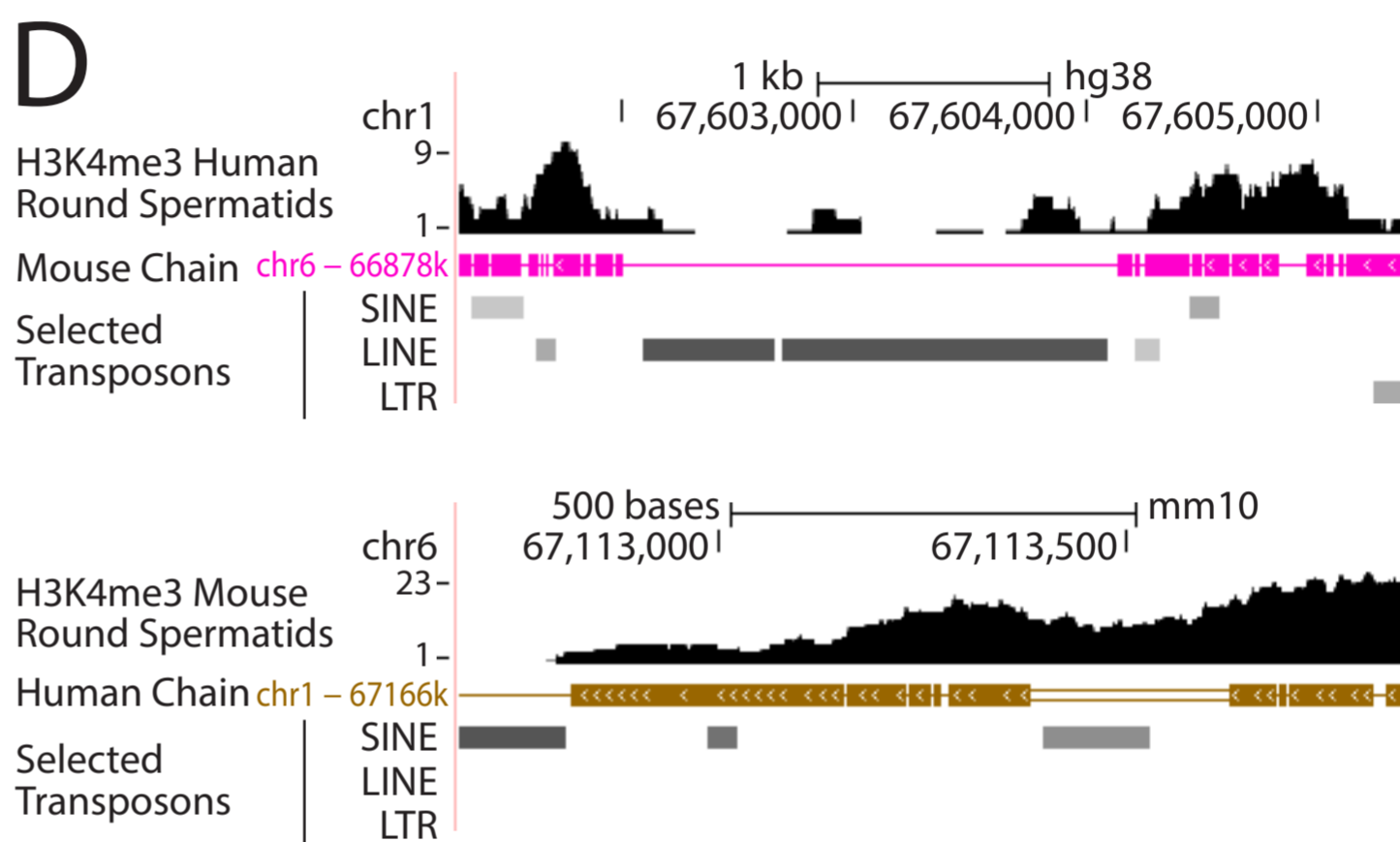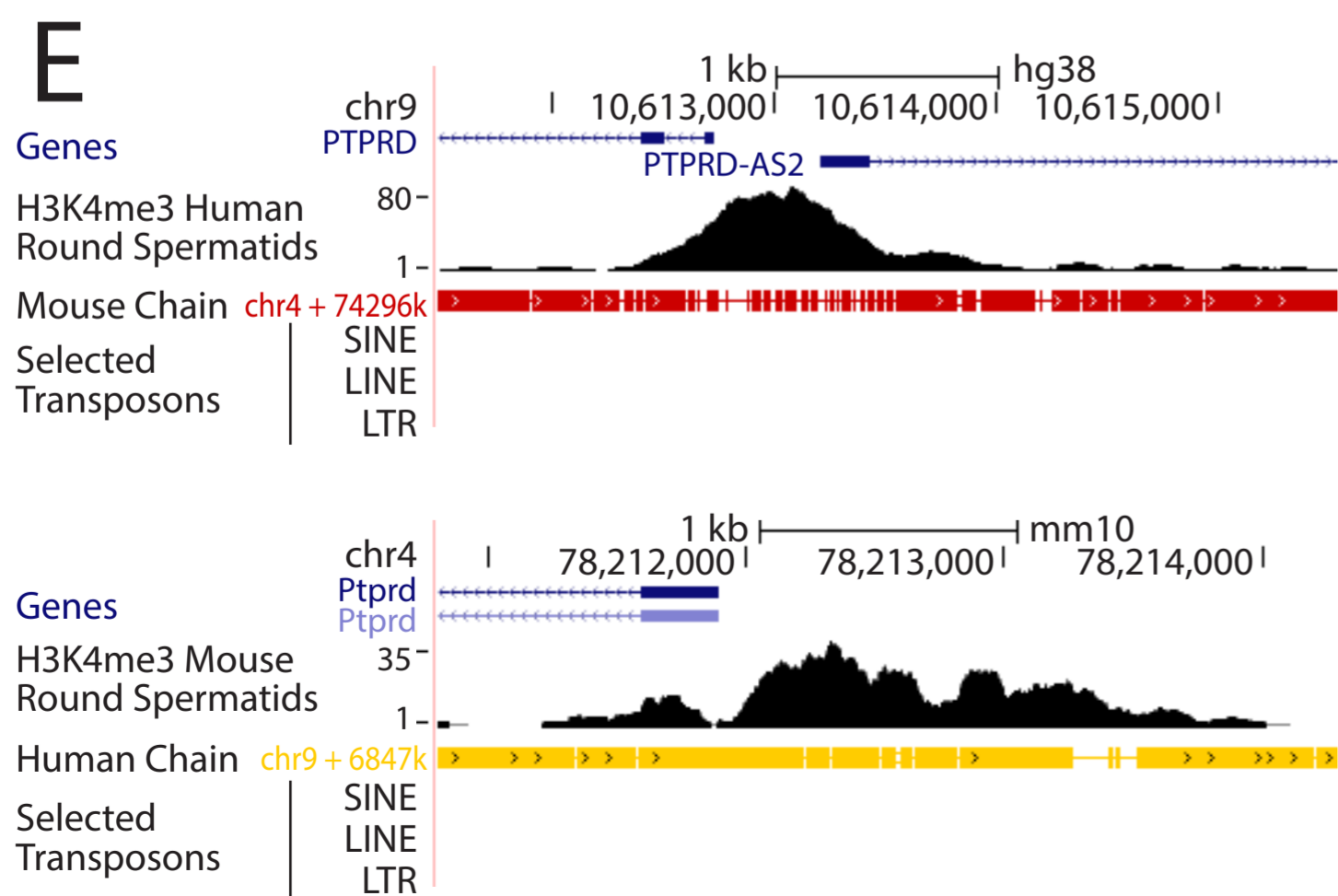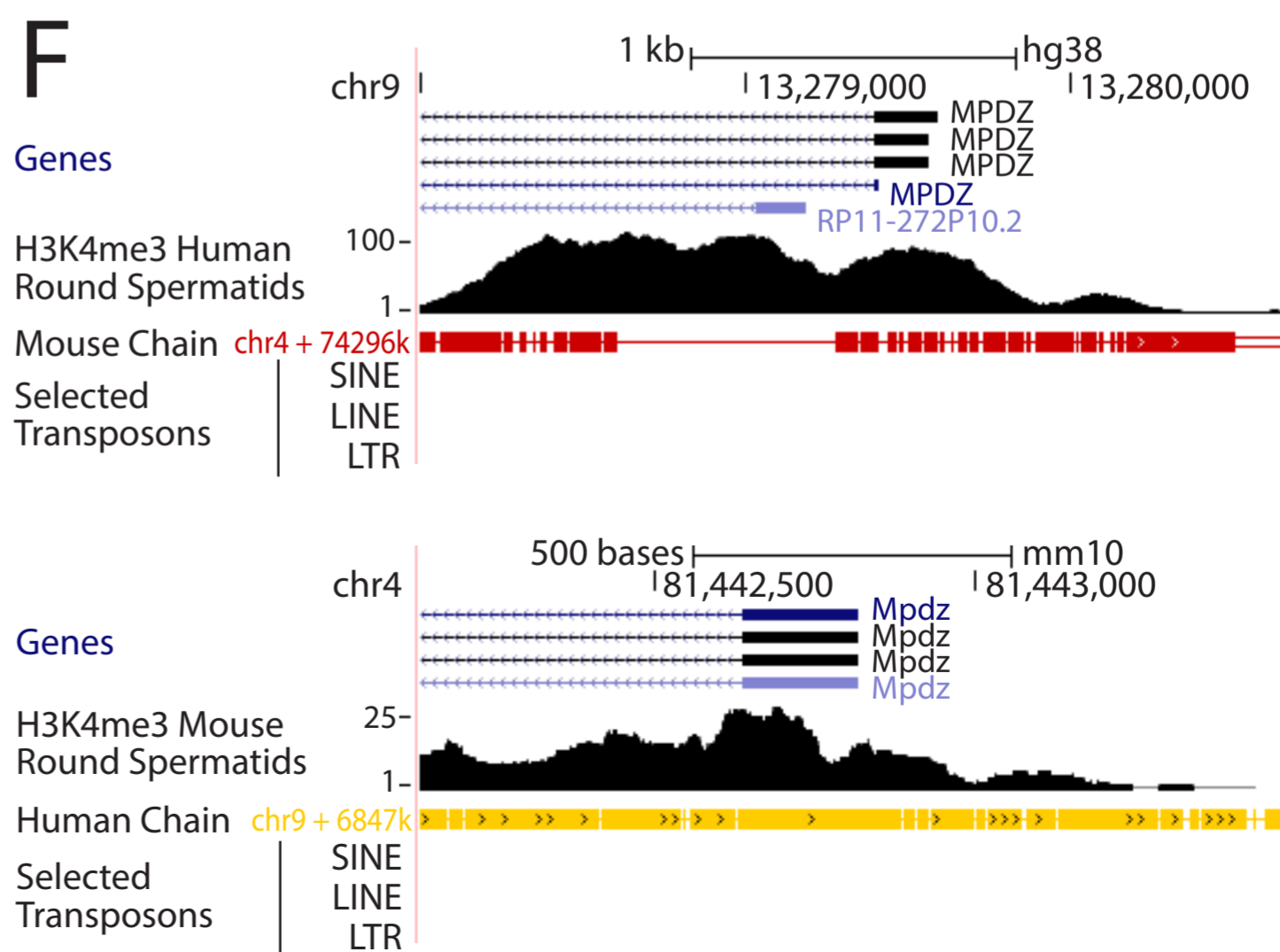

Supplement: S11 Fig — (A) Proportions of human-mouse homologous regions classified into each model. (B) Scatterplot of all homologous regions showing the degree of dependence to indels (rind, x axis) versus the degree of dependence to substitutions (rmut, y axis). Actual data for selected homologous regions (red circles) were given in panel (C)—(F). (C-D): Examples of regions with local-sequence-dependent H3K4me3 changes, within which the insertions of LINE-L1 transposons are associated with H3K4me3 loss in mouse and human, respectively. (E-F): Examples of regions with local-sequence-independent H3K4me3 variations. (E): The H3K4me3 variation is independent of both substitutions and indels. (F): The H3K4me3 variation is independent of large sequence insertions. (PDF) [file pcbi.1006673.s011.pdf]
