# Supplementary material for: A likelihood approach to testing hypotheses on the co-evolution of epigenome and genome
Source: PLoS Comput Biol. 2018 Dec 26;14(12):e1006673. doi: 10.1371/journal.pcbi.1006673 (PMC6324829; doi:10.1371/journal.pcbi.1006673)
Supplement: S7 Fig — Log odds ratio > 0 or < 0 corresponds to an increased or decreased level of enrichment. Error bars represent 95% confidence interval of log odds ratios. *: p-value of chi-square test < 0.05. **: p-value of chi-square test < 0.01. ***: p-value of chi-square test < 0.001. (PDF) [file pcbi.1006673.s007.pdf]

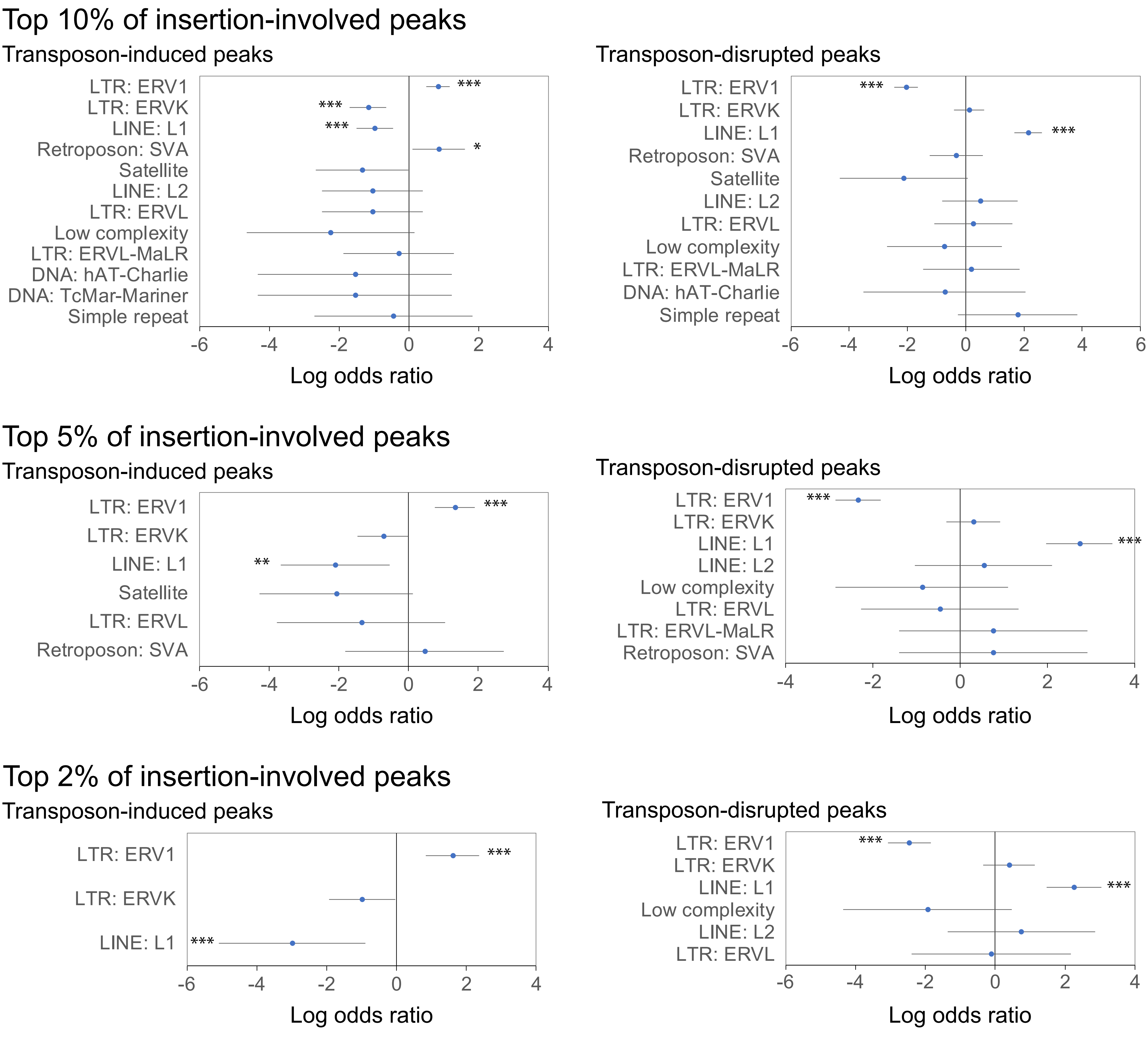

Top 10% of insertion-involved peaks
Transposon-induced peaks
LTR: ERV1
LTR: ERVK
LINE: L1
Retroposon: SVA
Satellite
LINE: L2
LTR: ERVL
Low complexity
LTR: ERVL-MaLR
DNA: hAT-Charlie
DNA: TcMar-Mariner
Simple repeat
***
***
***
*
-6 -4 -2 0 2 4
Log odds ratio
Transposon-disrupted peaks
LTR: ERV1
LTR: ERVK
LINE: L1
Retroposon: SVA
Satellite
LINE: L2
LTR: ERVL
Low complexity
LTR: ERVL-MaLR
DNA: hAT-Charlie
Simple repeat
***
***
-6 -4 -2 0 2 4 6
Log odds ratio
Top 5% of insertion-involved peaks
Transposon-induced peaks
LTR: ERV1
LTR: ERVK
LINE: L1
Satellite
LTR: ERVL
Retroposon: SVA
***
**
-6 -4 -2 0 2 4
Log odds ratio
Transposon-disrupted peaks
LTR: ERV1
LTR: ERVK
LINE: L1
LINE: L2
Low complexity
LTR: ERVL
LTR: ERVL-MaLR
Retroposon: SVA
***
***
-4 -2 0 2 4
Log odds ratio
Top 2% of insertion-involved peaks
Transposon-induced peaks
LTR: ERV1
LTR: ERVK
LINE: L1
***
***
-6 -4 -2 0 2 4
Log odds ratio
Transposon-disrupted peaks
LTR: ERV1
LTR: ERVK
LINE: L1
Low complexity
LINE: L2
LTR: ERVL
***
***
-6 -4 -2 0 2 4
Log odds ratio
