# Supplementary material for: A likelihood approach to testing hypotheses on the co-evolution of epigenome and genome
Source: PLoS Comput Biol. 2018 Dec 26;14(12):e1006673. doi: 10.1371/journal.pcbi.1006673 (PMC6324829; doi:10.1371/journal.pcbi.1006673)
Supplement: S8 Fig — (A) Demonstration of a L1-disrupted H3K4me3 peak. Shaded bands between two species indicate homologous sequences. Sequence with hatch pattern: insertion of a LINE-L1 transposon in Species 1, which was excluded in the analyses. (B) Log fold changes of H3K4me3 ChIP-Seq read counts between regions without L1 insertions and their corresponding homologous regions. (C) Log H3K4me3 ChIP-Seq intensities within regions without L1 insertions (left column) and flanking regions of the L1 insertions (right column). (PDF) [file pcbi.1006673.s008.pdf]

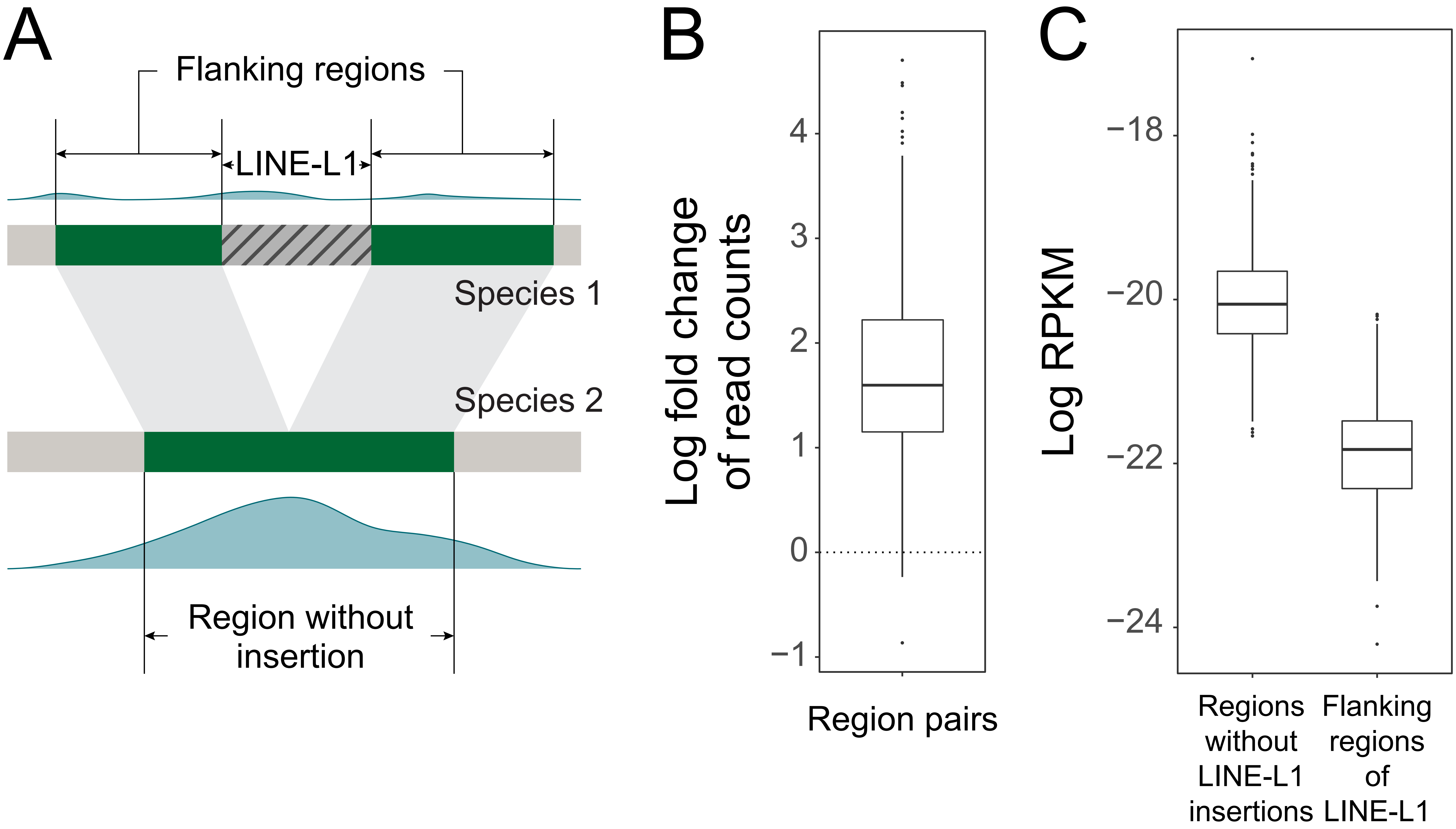

A
Flanking regions
LINE-L1
Species 1
Species 2
Region without insertion
B
Log fold change of read counts
4
3
2
1
0
−1
Region pairs
C
Log RPKM
−18
−20
−22
−24
Regions without LINE-L1 insertions
Flanking regions of LINE-L1
