# Supplementary material for: A likelihood approach to testing hypotheses on the co-evolution of epigenome and genome
Source: PLoS Comput Biol. 2018 Dec 26;14(12):e1006673. doi: 10.1371/journal.pcbi.1006673 (PMC6324829; doi:10.1371/journal.pcbi.1006673)
Supplement: S12 Fig — (A) Counts of different transposon families within different types of peak (B) Odds ratios between different transposon families and transposon-involved peaks. Log odds ratio > 0 or < 0 corresponds to an increased or decreased level of enrichment. Error bars represent 95% confidence interval of log odds ratios. *: p-value of chi-square test < 0.05. **: p-value of chi-square test < 0.01. ***: p-value of chi-square test < 0.001. (PDF) [file pcbi.1006673.s012.pdf]

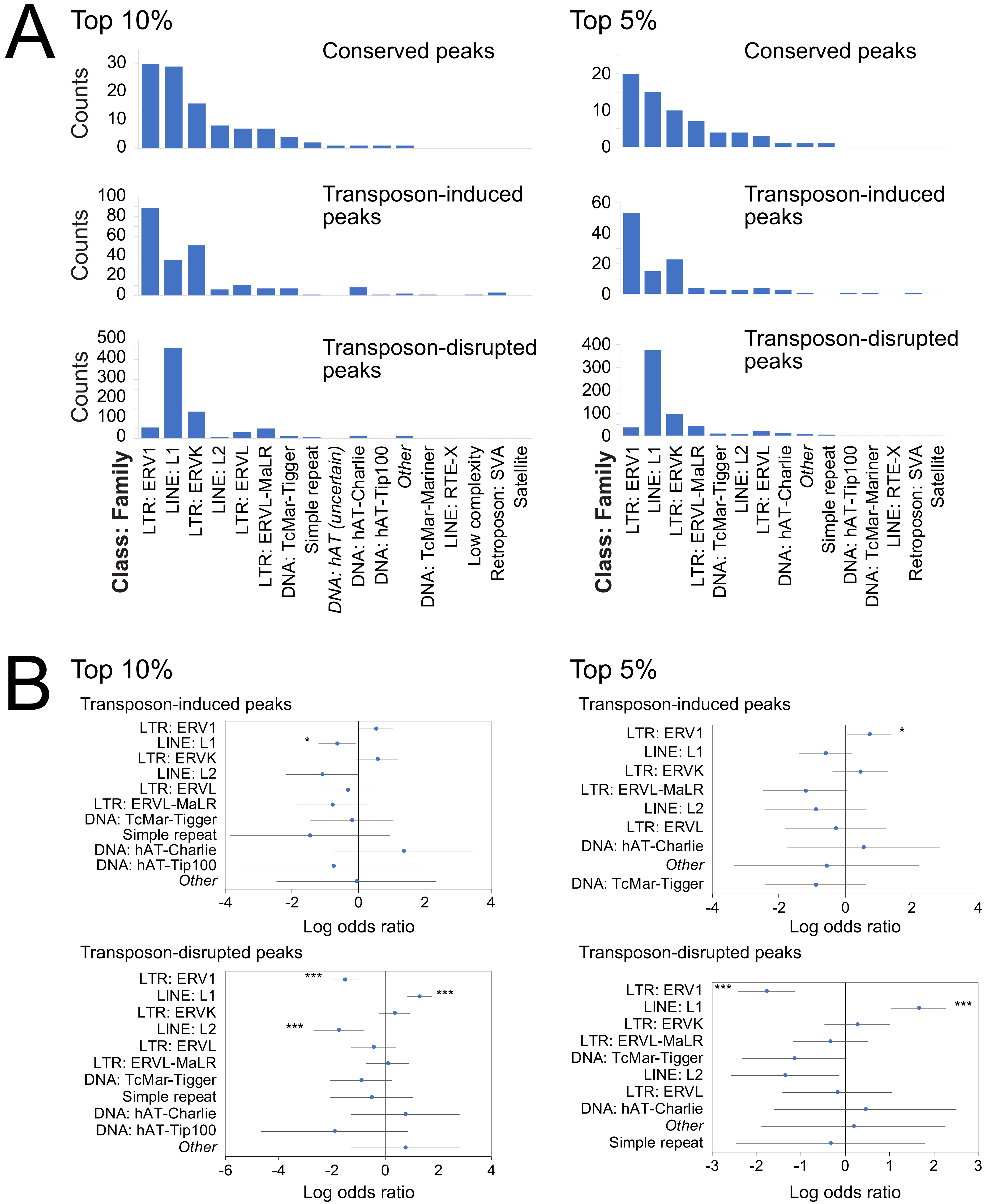
A
Top 10%
Conserved peaks
Counts
Transposon-induced peaks
Transposon-disrupted peaks
Class: Family
LTR: ERV1
LINE: L1
LTR: ERVK
LINE: L2
LTR: ERVL
LTR: ERVL-MaLR
DNA: TcMar-Tigger
Simple repeat
DNA: hAT (uncertain)
DNA: hAT-Charlie
DNA: hAT-Tip100
Other
DNA: TcMar-Mariner
LINE: RTE-X
Low complexity
Retroposon: SVA
Satellite
Top 5%
B
Top 10%
Transposon-induced peaks
Log odds ratio
Transposon-disrupted peaks
Top 5%
Transposon-induced peaks
Transposon-disrupted peaks
Log odds ratio
