# Supplementary material for: A likelihood approach to testing hypotheses on the co-evolution of epigenome and genome
Source: PLoS Comput Biol. 2018 Dec 26;14(12):e1006673. doi: 10.1371/journal.pcbi.1006673 (PMC6324829; doi:10.1371/journal.pcbi.1006673)
Supplement: S1 Table — A total of 8 datasets (Simulation column) were simulated with corresponding parameters (Columns s, μ, and κ) under each model. Other parameters are given in Methods. (PDF) [file pcbi.1006673.s013.pdf]

S1 Table. Parameters used for simulation.

A total of 8 datasets (Simulation column) were simulated with corresponding parameters (Columns s, μ, and κ) under each model. Other parameters are given in Methods.

Simulation	s	μ	κ
**1**	0.01	0.1	0.05
**2**	0.1	0.1	0.05
**3**	1	0.1	0.05
**4**	0.5	0.01	0.05
**5**	0.5	0.1	0.05
**6**	0.5	0.1	0.01
**7**	0.5	0.1	0.1
**8**	0.5	0.1	1
