# Supplementary material for: A likelihood approach to testing hypotheses on the co-evolution of epigenome and genome
Source: PLoS Comput Biol. 2018 Dec 26;14(12):e1006673. doi: 10.1371/journal.pcbi.1006673 (PMC6324829; doi:10.1371/journal.pcbi.1006673)
Supplement: S2 Table — Three randomly sampled subsets of all homologous region pairs were used to estimate parameters for all four models. Parameters remained close to each other among all subsets. (PDF) [file pcbi.1006673.s014.pdf]

S2 Table. Evolutionary parameters estimated from sampled regions.
Three randomly sampled subsets of all homologous region pairs were used to estimate parameters for all four models. Parameters remained close to each other among all subsets.

	Sampled subset	π_1	s	μ	κ
**Model N**	#1	0.0694	0.0717	0.0478	0.7695
	#2	0.0675	0.0731	0.0457	0.7732
	#3	0.0667	0.0715	0.0502	0.7509
**Model I**	#1	0.0694	0.0733	0.0464	0.7498
	#2	0.0675	0.0745	0.0444	0.7374
	#3	0.0667	0.0731	0.0487	0.7500
**Model M**	#1	0.0694	0.0700	0.0491	0.7392
	#2	0.0675	0.0715	0.0469	0.7434
	#3	0.0667	0.0699	0.0515	0.7202
**Model B**	#1	0.0694	0.0731	0.0465	0.7501
	#2	0.0675	0.0743	0.0445	0.7356
	#3	0.0667	0.0729	0.0488	0.7494
