# Supplementary material for: A likelihood approach to testing hypotheses on the co-evolution of epigenome and genome
Source: PLoS Comput Biol. 2018 Dec 26;14(12):e1006673. doi: 10.1371/journal.pcbi.1006673 (PMC6324829; doi:10.1371/journal.pcbi.1006673)
Supplement: S3 Table — (PDF) [file pcbi.1006673.s015.pdf]

S3 Table. Parameters estimated using different MACS2 q-value cutoffs.

MACS2 peak calling q-value cutoff	π_1	s	μ	κ
**0.1 (default)**	0.067	0.0745	0.0444	0.737
**0.05**	0.059	0.0745	0.0444	0.721
**0.01**	0.05	0.0745	0.0444	0.656
