# Supplementary material for: A likelihood approach to testing hypotheses on the co-evolution of epigenome and genome
Source: PLoS Comput Biol. 2018 Dec 26;14(12):e1006673. doi: 10.1371/journal.pcbi.1006673 (PMC6324829; doi:10.1371/journal.pcbi.1006673)
Supplement: S4 Table — (PDF) [file pcbi.1006673.s016.pdf]

S4 Table. Number of insertion-involved homologous region pairs selected with different r_{ind} percentage cutoffs.

	Percentage cutoff for $\lvert r_{ind} \rvert$		
	**2%**	**5%**	**10%**
**Sequence-dependent regions**	829	2,070	4,140
**Sequence-independent**	1,819	4,546	9,092
